# Approaches to Improve the Quantitation of Oxytocin in Human Serum by Mass Spectrometry

**DOI:** 10.3389/fchem.2022.889154

**Published:** 2022-06-09

**Authors:** Anke Hering, Beverly Jieu, Alun Jones, Markus Muttenthaler

**Affiliations:** ^1^ Institute for Molecular Bioscience, The University of Queensland, Brisbane, QLD, Australia; ^2^ Institute of Biological Chemistry, Faculty of Chemistry, University of Vienna, Vienna, Austria

**Keywords:** oxytocin, LC-MS, neuropeptide, analytical method development, sample preparation

## Abstract

The neuropeptide oxytocin (OT) regulates several peripheral and central functions and is a molecule of interest in psychiatric diseases such as autism spectrum disorder, schizophrenia, anxiety and depression. The study of OT in human serum samples is however hampered by inconsistent sample preparation and analysis as well as low endogenous blood concentration (1–10 pM). This results in varying reports on OT’s blood levels and interpretation of OT’s role in different (patho)physiological states. Quantitative mass spectrometry (MS) is a highly promising technology to address this problem but still requires large sample volumes to achieve adequate sensitivity and reliability for the quantitation of compounds at low concentrations. We therefore systematically evaluated sample preparation methods for MS to achieve a reliable sample preparation protocol with good peptide recovery, minimal matrix effects and good overall method efficiency in line with FDA guidelines for bioanalytic method development and validation. Additionally, we investigated a strategy to improve the ionization efficiency of OT by adding charged and/or hydrophobic moieties to OT to improve the lower limit of quantitation. Optimized sample preparation in combination with OT modification with a quaternary pyridinium ion improved the sensitivity of OT by ∼40-fold on a tandem triple quadrupole mass spectrometer (API4000 QTRAP), resulting in a lower limit of quantitation of 5 pM in water (linear range 5 pM – 1 mM) and 2 nM in human serum (linear range 2 nM – 1 mM) compared to 200 pM in water and 86 nM in serum with unmodified OT. This approach and protocol provide a solid foundation towards method development for OT quantitation using MS, which should be of high value for fundamental research as well as clinical monitoring of OT upon drug treatments.

## Introduction

Oxytocin (OT) is an important neuropeptide and peptide hormone mediating several physiological functions. These range from peripheral functions like muscle contractions in the uterus during birth (Gimpl and Fahrenholz, 2001; Leng et al., 2008; Jurek and Neumann, 2018), ejaculation (Hib, 1974a; Hib, 1974b; Hib, 1977) and milk-ejection (McNeilly et al., 1983; Nishimori et al., 1996; Erickson et al., 2020), as well as anti-inflammatory effects in the cardiovascular (Jovanovic et al., 2019; Meusel et al., 2021) and gastrointestinal systems (Pfister et al., 2005; Welch et al., 2009; Welch et al., 2014; Tang et al., 2019), to central nervous system functions such as maternal care, pair-bonding, empathy, memory and learning (Heinrichs et al., 2009; Rasie Abdullahi et al., 2018; Salighedar et al., 2019). OT holds therapeutic potential for a wide variety of diseases including breast cancer (Ariana et al., 2018; Khori et al., 2018; Liu et al., 2020), cardiovascular disease (Merz et al., 2020), diabetes (Garrido-Urbani et al., 2018; Szeto et al., 2020; Yuan et al., 2020) and neurological disorders such as autism and schizophrenia (Green et al., 2001; Cyranowski et al., 2008; Goldman et al., 2008; Kéri et al., 2009; Feifel et al., 2010; Guastella et al., 2010; Bartholomeusz et al., 2015; Feifel et al., 2016; Alvares et al., 2017; Bradley and Woolley, 2017; Ooi et al., 2017). OT has also been studied for its potential as a biomarker, but progress has been hampered by inconsistent analysis methods and difficulties with reliably measuring the low levels of endogenous OT in the blood (1–10 pM) (Kagerbauer et al., 2013; Leng and Sabatier, 2016), resulting in contradicting measurements and rendering data interpretation difficult (Glovinsky et al., 1994; Bell et al., 2006; Jansen et al., 2006; Ozsoy et al., 2009; Miller et al., 2013; Strauss et al., 2015; Saxbe et al., 2019).

Radio- and enzyme-linked immunoassays are the most commonly used methods for OT analysis in biological samples, but they have been questioned for their lack of sensitivity and specificity as well as for their inconsistent sample preparation protocols (Szeto et al., 2011; McCullough et al., 2013; Robinson et al., 2014; Leng and Sabatier, 2016). Mass spectrometry (MS) is a promising alternative since it is a sensitive and mass-specific analytical method that can quantify a target analyte by its defined mass and fragmentation pattern, thereby excluding any metabolites that might still give a signal using immunoassays (McCullough et al., 2013). MS methods have been developed for OT quantitation using triple quadrupole (Zhang et al., 2011; Brandtzaeg et al., 2016) or orbitrap (Franke et al., 2019) mass spectrometers. However, achieving the required lower limit of quantitation (LLOQ) to reliably measure OT in the range of 1–10 pM remains challenging. We therefore set out to develop improved MS-based strategies and protocols towards the detection and quantitation of OT in biological samples.

The first challenge, one often overlooked, is that MS is susceptible to sample matrix interferences that cause ion suppression or enhancement, known as matrix effects (Mei et al., 2003; Panuwet et al., 2016). Matrix effects and their impact on analyte ionization depend on ionization type, biological matrix, chromatographic conditions, and sample preparation (Dams et al., 2003; Panuwet et al., 2016). Appropriate sample preparation is particularly important in reducing matrix effects as it removes impurities and matrix interferences (Szeto et al., 2011; Christensen et al., 2014; Robinson et al., 2014; Lefevre et al., 2017). Most sample preparation protocols developed for OT analysis unfortunately do not evaluate such matrix effects and lack adequate characterization according to the US Food and Drug Administration (FDA) guidelines for bioanalytical method development (Kramer et al., 2004; Brandtzaeg et al., 2016; Franke et al., 2019). The second challenge is that MS sensitivity is still not good enough to reliably quantify OT’s low endogenous levels, requiring large sample volumes to achieve adequate sensitivity or specialized equipment not available in most laboratories (e.g., 2D LC-MS/MS) (Dams et al., 2003; Zhang et al., 2011; Liu et al., 2019). When using electrospray ionization MS (ESI-MS), the sensitivity relies heavily on the analyte’s ionization efficiency and ion transmission (Kebarle and Tang, 1993; Page et al., 2007). These factors can be improved by introducing derivatizations to the peptide such as hydrophobic and charged amino acids, as well as quaternary ammonium, phosphonium, imidazolium and pyridinium salts (Bąchor et al., 2014; Qiao et al., 2014; Qiao et al., 2015; Waliczek et al., 2016).

Here, we describe the development of a robust sample preparation protocol following FDA guidelines in terms of LLOQ (Administration, U.F.A.D., 2018) and an OT derivatization method to improve OT’s ionization efficiency with the aim to improve the overall sensitivity of detecting and quantifying OT in biological samples.

## Results

### Method Development

OT was synthesized by manual Fmoc solid phase peptide synthesis (Kremsmayr and Muttenthaler, 2022). The exact concentration and purity of the peptide was determined *via* RP-HPLC (Supplementary Table S1) and compared against two peptide standards of known concentration.

#### Analyte Adsorption to HPLC Inserts

**FIGURE 1:**
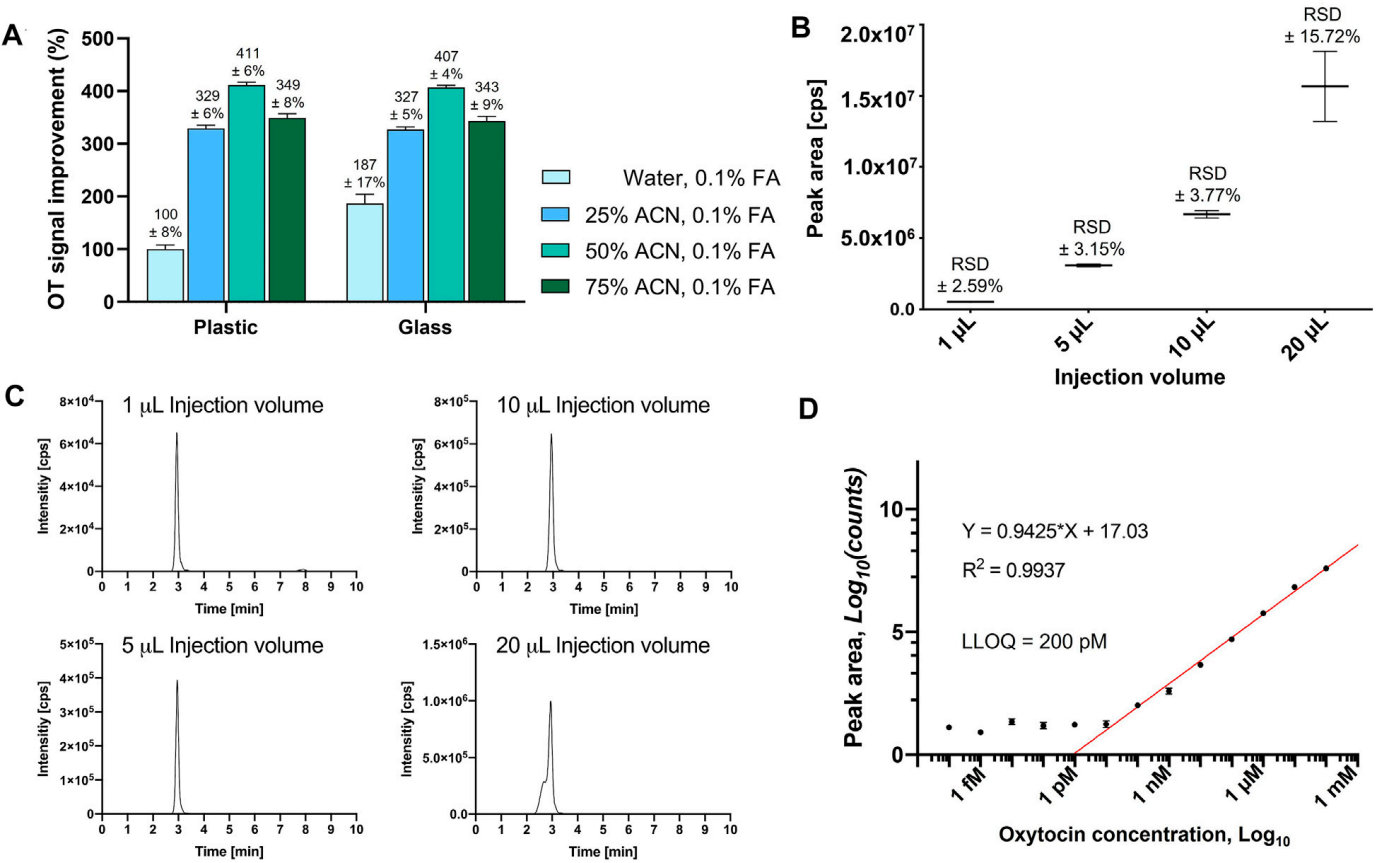
Evaluation of HPLC vial adsorption, optimal injection volume and LLOQ of OT on an API4000 QTRAP. **(A)** OT MS peak area signal improvement of a 10 µM OT solution with different ACN concentrations from either plastic or glass HPLC inserts after 24 h, normalized to the signal of the OT solution in water, 0.1% FA, from a plastic HPLC insert. Results are presented as the mean percentage improvement ±SD; *n* = 3. **(B)** Evaluation of different injection volumes (1, 5, 10, and 20 μL) of an OT standard solution (10 μM in 50% ACN_aq_/0.1% FA). The values are presented as mean ± RSD of four consecutive injections (*n* = 4). **(C)** Representative LCMS traces for the injection volumes to visualize peak shapes. **(D)** OT standard curve to determine LLOQ.

Adsorption of the analyte to sample preparation materials (e.g., HPLC inserts) can result in inconsistent and inaccurate measurements (Goebel-Stengel et al., 2011). Addition of organic solvent (e.g., ACN) can prevent this adsorption and improve ionization during MS analysis (Sterner et al., 2000; Gosetti et al., 2010). We therefore prepared OT standards (10 µM) with different ACN concentrations (H_2_O, 25, 50, 75% ACN) and analyzed them in plastic polypropylene or silanized glass HPLC inserts in three independent experiments every hour for 24 h. An API4000 QTRAP MS instrument tuned to OT and equipped with an Agilent C_18_ column was used for this study (Supplementary Table S2). The OT MS signal (peak area) was twice as large in glass inserts than in plastic inserts (10 µM OT in H_2_O/0.1% formic acid, FA) (Figure 1A). The use of ACN as the organic co-solvent enhanced the MS signal for OT 3–4-fold in both plastic and glass inserts, with no significant differences between plastic and glass inserts. The best signal was obtained in 50% ACN_aq_/0.1% FA, which was selected for the rest of the study.

#### Injection Volume

We evaluated 1, 5, 10, and 20 µL injection volumes of a standard OT solution (10 μM, 50% ACN_aq_/0.1% FA) by LC-MS/MS (Figure 1B). Injection volumes of 1–10 µL had sharp peaks, high signal-to-noise ratio, and low variability (relative standard deviation (RSD) ± 2.59–3.77%), while injection volumes of 20 µL displayed broader peaks with a shoulder and higher variability (RSD ±15.72%) (Figure 1C). A 10 µL injection volume was therefore used for the rest of this study.

#### Standard Curve and Lower Limit of Quantitation

The linear range was determined by the maximum number of points that could be included for the R^2^ coefficient to remain ≥0.9. LLOQ was determined by visual evaluation of the calibration curve (smallest value on the linear range, before the plateau) and should be at least ≥ ×5 of the blank signal (Supplementary Figure S2) (Shrivastava and Gupta, 2011). A dilution series of OT ranging from 0.1 mM to 0.1 fM in 50% ACN_aq_/0.1% FA was prepared and analyzed on an API4000 QTRAP (Figure 1D). The calibration curve displayed a plateau from 0.1 fM to 100 pM OT and formed a linear range from 1 nM to 0.1 mM (R^2^ = 0.9971). 200 pM was the lowest value lying on the linear range before the plateau and thus determined as the LLOQ of OT.

#### Sample Preparation Methods and Parameters

**FIGURE 2:**
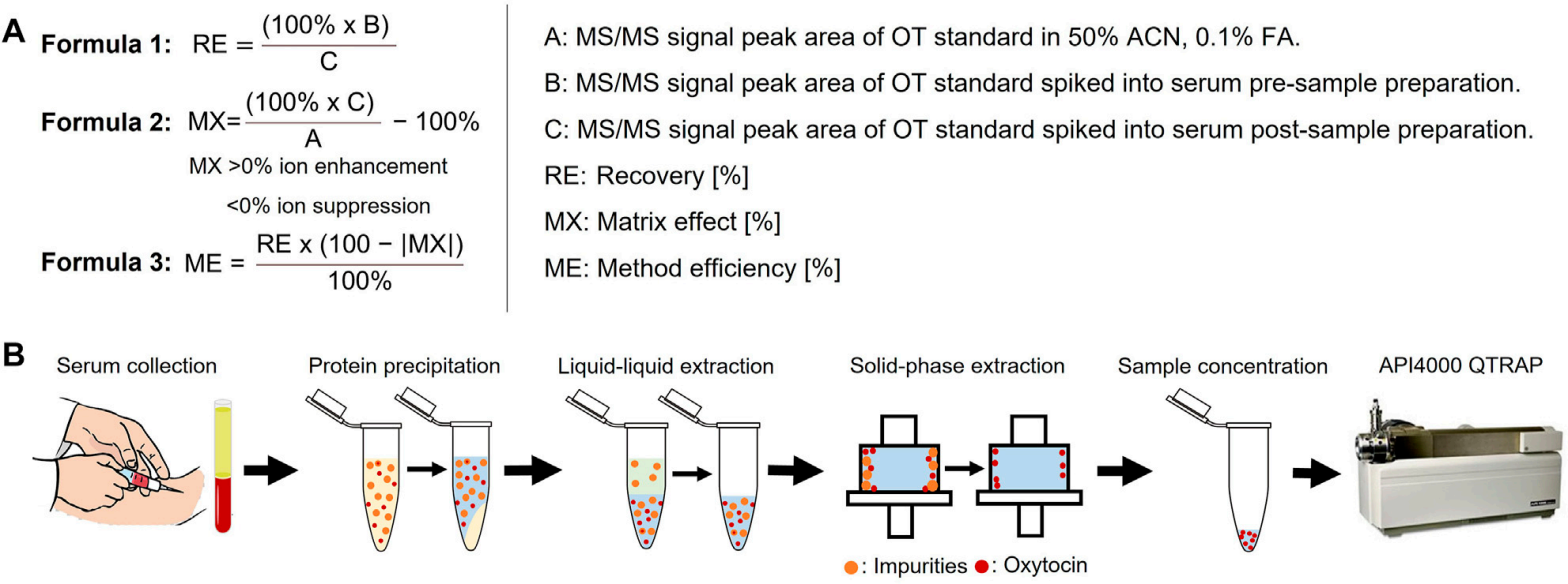
Sample preparation flowchart and analysis. **(A)** Calculation of peptide recovery (RE), matrix effects (MX) and overall method efficiency (ME). RE provides information on the peptide recovery of the sample preparation protocol (from protein precipitation to sample concentration). MX provides information on observed matrix effects, with positive values indicating ion enhancement and negative values indicating ion suppression effects. ME combines RE and MX values to provide comparative information on the overall method efficiency. RE values >115% and MX values >±100% were set to zero for the ME calculation to exclude methods based on false positives. MS/MS signal peak areas are taken as log_10_ (counts). **(B)** Overview of the sample preparation steps that were optimized.

Sample preparation is a critical step to reduce sample matrix interferences that affect the accuracy, precision and robustness of MS analysis (Panuwet et al., 2016). Six different sample preparation protocols (Table 1) were designed and evaluated regarding the matrix effect (MX), peptide recovery (RE), and overall method efficiency (ME) *via* the pre- and post-spike method (Matuszewski et al., 2003) by spiking serum at three OT concentrations (0.1, 10, and 50 µM) to give a final OT concentration of 4.5 µM, 0.91 µM and 9.09 nM. Briefly, the pre-and post-spike method involves (A) spiking a blank solution (50% ACN_aq_/0.1% FA) with an OT standard, (B) spiking a serum sample with OT before sample preparation, and (C) spiking a serum sample with OT after sample preparation. Samples were injected (10 µL) into the API4000 QTRAP and peak areas were analyzed. The calculation of the RE, MX, and ME values and the overview of the sample preparation are described in Figure 2.

**TABLE 1:** Overview of tested sample preparation protocols for OT analysis in human serum. Modified protocol parameters are bolded. RE values >115% were set to 0 for ME calculations. Results for protocols are presented qualitatively as a heat map with green, yellow and red representing good, average and poor values respectively. Exact values with errors and cut-off ranges can be found in Supplementary Table S3.

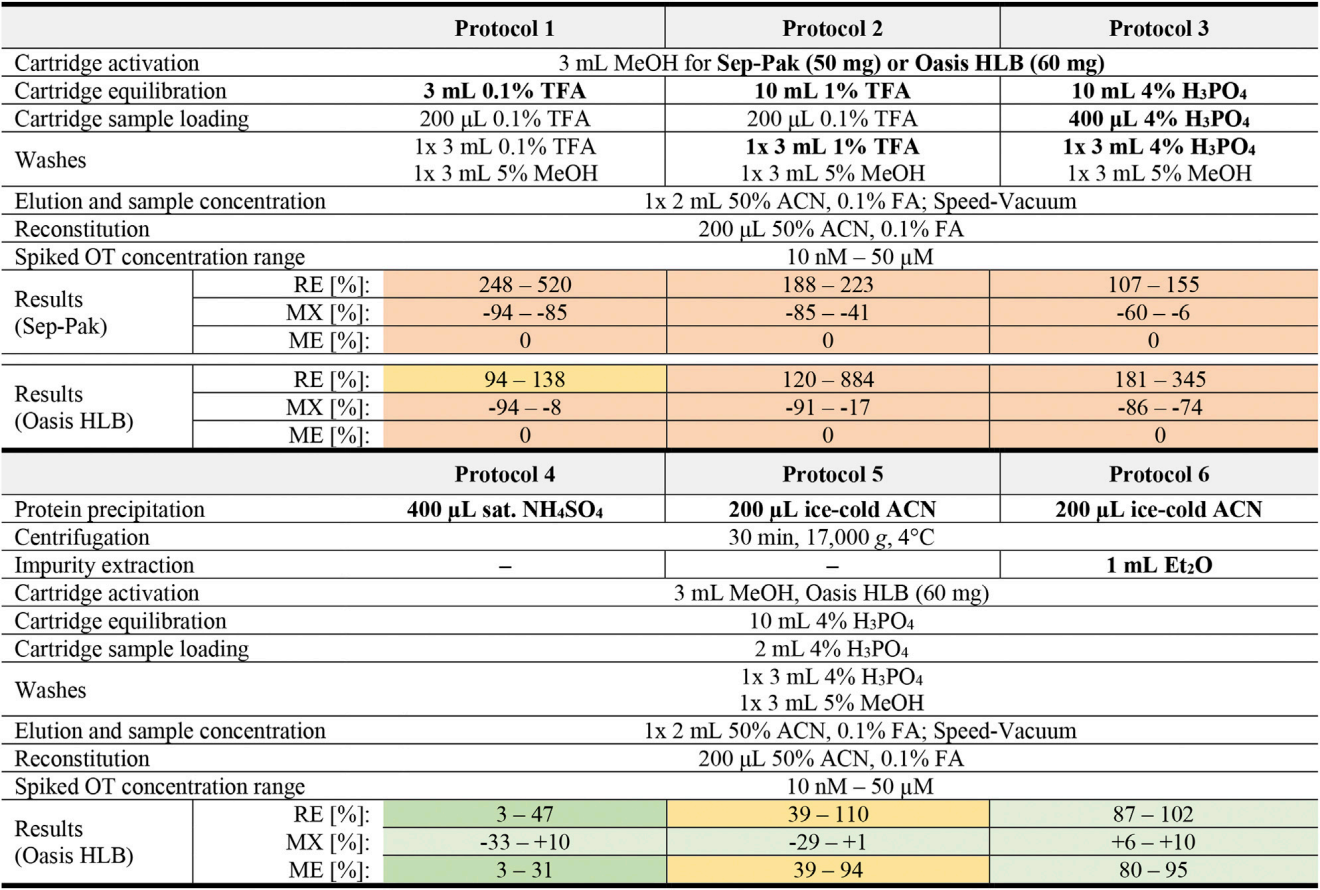

With Protocols 1, 2, and 3, we evaluated two acids commonly used for solid phase extraction, namely TFA (trifluoroacetic acid, 0.1 and 1%) and H_3_PO_4_ (phosphoric acid, 4%). We evaluated these protocols on two solid phase extraction cartridges, the Sep-Pak (50 mg) and Oasis Hydrophilic-Lipophilic-Balanced (HLB, 60 mg) cartridges (Table 1). A continuous pressure increase on the LC-MS/MS (API4000 QTRAP) was observed during analysis of samples prepared with Sep-Pak cartridges, indicating incomplete sample clean-up and a consequent accumulation of impurities on the HPLC column. Oasis HLB cartridges were therefore selected for the sample preparation step. Of the three protocols tested on Oasis HLB cartridges, Protocol 3 was selected for further method development since the MX values were the most consistent across the measured OT concentrations (Supplementary Table S3).

To improve sample clean-up and reduce matrix effects, an additional sample clean-up step prior to solid phase extraction was introduced. A saturated NH_4_SO_4_ solution (Protocol 4) or ice-cold ACN (Protocol 5 and 6) were used to precipitate larger serum proteins, leaving OT in the supernatant (Protocol 4: 1.25 µM–2.5 nM OT in 400 µL NH_4_SO_4_ solution; Protocol 5 & 6: 2.5 µM–5 nM OT in 200 µL ACN). The precipitated impurities were centrifuged into a pellet and removed. An additional liquid-liquid extraction step was introduced in Protocol 6 to further remove hydrophobic compounds. In this step, ice-cold diethyl ether (Et_2_O) was mixed with the supernatant after centrifugation, extracting hydrophobic molecules into the ether phase, leaving OT in the aqueous phase.

Protein precipitation with saturated NH_4_SO_4_ (Protocol 4) resulted in low OT recovery (3–47%), possibly due to co-precipitation of OT and serum proteins. Precipitation with cold ACN (Protocol 5) or cold ACN combined with Et_2_O liquid-liquid extraction (Protocol 6) worked well with OT recoveries in the 39–110% and 87–102% range, respectively. Particularly the ACN precipitation with Et_2_O liquid-liquid extraction resulted in substantial reduction of matrix effects across all concentration tested (MX <±12%). The resulting ME for Protocols 4, 5 and 6 were 3–31%, 39–94%, and 82–95% across the tested concentrations, and Protocol 6 was therefore selected for further characterization.

#### Evaluation of Reconstitution Volume of Sample Preparation Protocol

Decreasing the reconstitution volume increases the analyte’s concentration and thereby its detection limits, but this can also lead to more pronounced matrix effects. To determine the RE, MX, and ME of different reconstitution volumes, serum samples were spiked with 50 µM OT *via* the pre- and post-spike method and prepared using Protocol 6. Samples were reconstituted in four different volumes (40, 60, 80, and 100 μL; 50% ACN_aq_/0.1% FA) and evaluated compared to the initial reconstitution volume of 200 µL. Changing the reconstitution volume also affects the analyte’s concentration, thus altering the LLOQ of the method. Hence, human serum samples were spiked with OT standards (0.1 pM–1 mM), prepared using Protocol 6, and reconstituted in 40, 60, 80 or 100 μL; 50% ACN_aq_/0.1% FA). Samples were analyzed in the API4000 QTRAP and a standard curve was determined for each reconstitution volume. While reconstitution volumes of both 60 and 200 µL had good RE, MX, and ME results, a 60 µL reconstitution volume yielded a ∼2-fold improvement in LLOQ as compared to the initially used 200 µL (Supplementary Table S4). 60 µL reconstitution volume was thus chosen for the final protocol (Protocol 6′). RE, MX, and ME of the final Protocol 6′ was determined *via* the pre- and post-spike method with three OT concentrations (50 , 10 and 0.1 µM). This gave a MX of -16–-10%, 81–87% RE, 71–77% ME and a LLOQ of 86 ± 17 nM in human serum (Supplementary Figure S3, Supplementary Table S5). Protocol 6′ was used for further method validation.

#### Method Validation: Precision and Accuracy

To determine precision and accuracy of the method, human serum was spiked with OT standard to give final serum OT concentrations of 1.5, 10 and 40 µM. The spiked human serum samples were purified with Protocol 6’ and analyzed. The LCMS peak areas of these samples were compared against the OT standard curve to calculate the expected concentration present in serum (*n* = 5). Precision is expressed as a percentage of standard deviation from the mean value and accuracy as percentage error from the theoretical concentration. The intraday precision for the quantitation of OT in human serum was 6.4–7.6% and the intraday accuracy was -7.0–15.7% (Supplementary Table S6). The method therefore fulfills largely the FDA acceptance criteria for precision (±15%) and accuracy (±15%) for bioanalytical method validation (except for one intraday accuracy measurement of 15.7%) (Administration, U.F.A.D., 2018).

### Improving Sensitivity

To increase the sensitivity of our analytical method, we investigated different MS instruments and strategies to improve OT’s ionization efficiency.

#### Protocol Evaluation Across Other Mass Spectrometry Instruments

A dilution series of OT (1 fM to 0.1 mM in 50% ACN_aq_/0.1% FA) was analyzed across four quadrupole time-of-flight flight (QTOF) mass spectrometers (TripleTOF 6600, TripleTOF 5600, ×500R, QstarElite) and two triple quadrupole instruments (API4000 QTRAP, QTRAP6500) (Supplementary Figure S4). The sensitivity of each instrument was determined using the LLOQ of OT (50% ACN_aq_/0.1% FA). The triple quadrupole mass spectrometers, API4000 QTRAP (LLOQ: 100 ± 12 pM) and QTRAP6500 (LLOQ: 40 ± 3 pM) had greater sensitivity for OT compared to the TOF instruments. The superior sensitivity of triple quadrupoles over QTOF instruments was expected due to their additional quadrupole, allowing them to be tuned to a specific potential for detection of one specific ion. However, it also highlighted that further improvement is necessary before being able to quantitatively measure OT out of complex biological samples. We continued with the method development on the API4000 QTRAP due to limited access to the QTRAP6500.

#### Improving the Analyte’s Ionization Efficiency Through Derivatization

MS analyte detection sensitivity in ESI-MS can also be enhanced by improving the ionization efficiency of the analyte through modifications of its hydrophobicity and/or charge state (Konermann et al., 2013). During ionization in ESI-MS, hydrophobic analytes tend to sit on the outside of the ionization droplet, resulting in easier and more efficient ionization. Analytes with high proton affinity (e.g., Arg-containing peptides) also increase signal intensity in ESI-MS due to formation of pre-charged species.

We investigated two strategies: 1) addition of either charged or hydrophobic amino acids, and 2) addition of positively charged small molecule moieties. The API4000 QTRAP was tuned for the detection of the new peptide analogues and a calibration curve in water was prepared for each (Table 2). Removing the stabilizing disulfide bond on OT might also improve ionization; we therefore assessed the oxidized and reduced form of the new analogues. The hydrophobicity and charge of each analogue, oxidized and reduced, was compared against that of native OT (Table 2).

**TABLE 2:** LLOQ values of OT analogues in 50% ACN_aq_/0.1% FA. A dilution series of each peptide ranging from 1 fM to 1 mM in 50% ACN_aq_/0.1% FA was measured (*n* = 3) on the API4000 QTRAP. LLOQ was determined by visual examination of the calibration curve and ensuring the signal was ≥5x the blank. LLOQ values < 20 pM (more sensitive than OT) are highlighted in green, >200 pM in red, values between 20 and 200 pM in grey and the four sequences with the lowest LLOQ values in a darker shade of green. **(i)** 4-bromo-N,N,N-triethylbutan-1-amminium, **(ii)** 3-(4-bromobutyl)-1-butyl-1H-imidazol-3-ium, **(iii)** iodoacetamide, **(iv)** 1-(4-bromobutyl)-4-methylpyridin-1-ium, * indicates C-terminal amide.

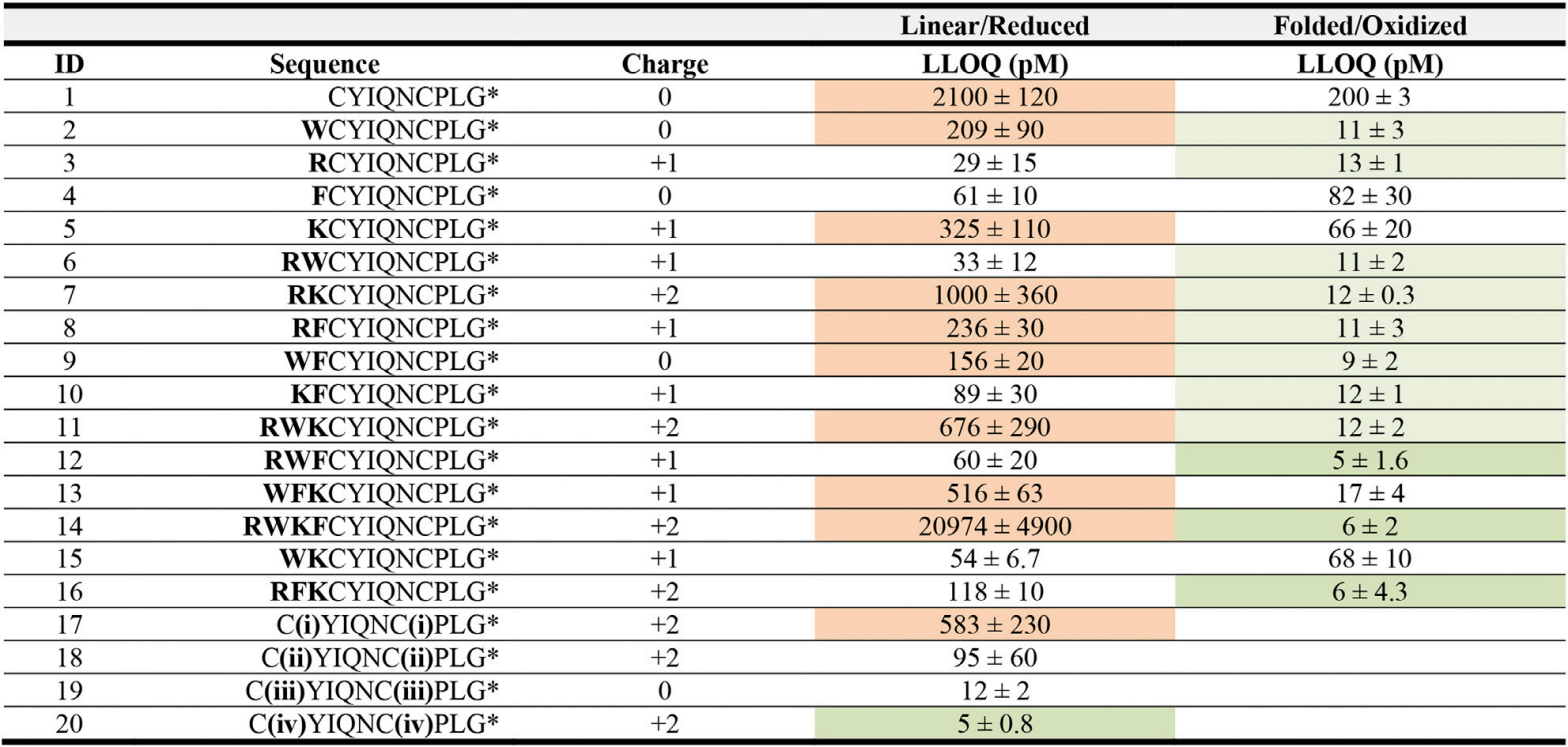

**FIGURE 3:**
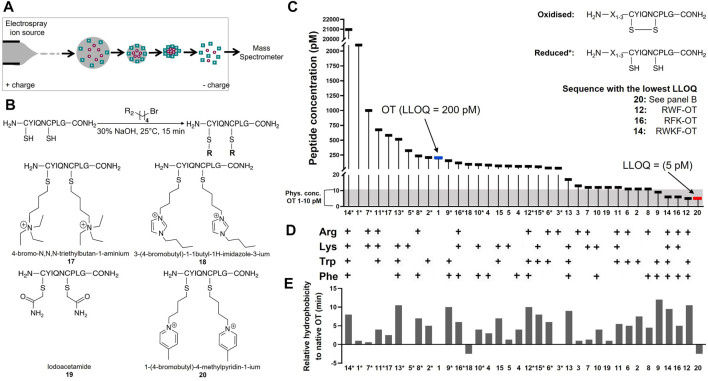
Improving MS sensitivity by modifying OT’s hydrophobicity and charge state. **(A)** Analytes form spherical droplets upon electrospray ionization in an ESI-MS. Hydrophilic analytes tend to remain more in the center of the ionization droplets, resulting in less efficient ionization compared to more hydrophobic analytes that tend to remain on the surface of the droplets. **(B)** Derivatization of OT with positively charged small molecule moieties. **(C)** LLOQs of synthesized and derivatized OT analogues. *Indicates reduced peptides. **(D)** Presence of amino acids Arg, Lys, Trp, and Phe in studied OT analogues. **(E)** Relative hydrophobicity on a C_18_-RP HPLC column compared to OT. Values > 0 indicate more hydrophobic peptides (longer retention time), while <0 indicate more hydrophilic peptides (shorter retention time) than OT.

In the first strategy, Arg, Lys, Trp, and Phe were added in different combinations to the N-terminus of reduced and folded/oxidized OT (Table 2, compounds **1–16**). The addition of single amino acids Arg, Lys, Trp and Phe yielded improved sensitivity (LLOQ of 13, 66, 11, and 82 pM respectively) compared to unmodified OT (200 pM); oxidized analogues generally yielded better sensitivity (Figure 3C). In particular, the Arg analogue (R-OT **3**, 13 pM) and Trp analogue (W-OT **2**, 11 pM) improved LLOQ in ESI-MS by 13–15-fold.

Addition of two amino acids to OT allowed the study of combinations of charged (Arg and Lys) and hydrophobic (Trp and Phe) amino acids. The addition of two charged or two hydrophobic residues did however not necessarily result in lower LLOQs than OT derivatives with a single residue addition. For example, WK-OT **15** (68 pM) had a relatively high LLOQ, and RK-OT **7** (12 pM) had a LLOQ similar to W-OT **2** (11 pM) and R-OT **3** (13 pM). In line with the results of the single residue derivatives, the combination of a charged amino acid (Arg/Lys) with a hydrophobic (Trp/Phe) improved the LLOQ in oxidized analogues but less so in reduced analogues. Overall, the addition of two amino acids improved the LLOQ by 13–16-fold for the oxidized analogues, with WF-OT **9** (9 pM) having the best sensitivity. These improvements were similar to the improvements of single amino acid derivatives, i.e., R-OT **3** (13 pM) and W-OT **2** (11 pM).

The addition of three amino acids allowed us to combine a charged residue with two hydrophobic residues and vice versa. The trend of the oxidized forms having better sensitivity than their reduced counterparts continued with these derivatives. Two of the best performing analogues, were RWF-OT **12** (5 pM) and RFK-OT **16** (6 pM). The addition of all four amino acids, RWKF-OT **14** (6 pM), resulted in a more hydrophobic peptide than native OT **1** (100 pM). From all analogues tested, the modifications RWF-OT **12** (5 pM), RFK-OT **16** (6 pM) and RWKF-OT **14** (6 pM) had the biggest impact on sensitivity, resulting in 20–25-fold improvement compared to the LLOQ of OT. Also, R-OT **3** (13 pM) and W-OT **2** (11 pM) should be mentioned since they are in a similar LLOQ range through the N-terminal addition of just a single amino acid.

For the second strategy, three small molecules, 2-bromo-N, N, N,-triethylethan-1-aminium (quaternary ammonium ion), 3-(4-bromobutyl)-1-butyl-1H-imidazol-3-ium (imidazolium ion) and 1-(4-bromobtyl)-4-methylpyridin-1-ium (pyridinium ion) were synthesized (Figure 3B) and used to derivatize the free Cys thiol groups of reduced OT (Figure 3B; **ID 17–20**) Iodoacetamide was also added to the free Cys thiol of reduced OT as another analogue. The LLOQ for the resulting OT analogues alkylated by (**i**) 4-bromo-N, N, N,-triethylbutan-1-aminium, (**ii**) 3-(4-bromobutyl)-1-butyl-1H-imidazol-3-ium, (**iii**) iodoacetamide and (**iv**) 1-(4-bromobutyl)-4-methylpyridin-1-ium were 583, 95, 12 and 5 pM respectively. The LLOQ of [1-(4-bromobutyl)-4-methylpyridin-1-ium)_2_-OT (**20**) was 25-fold lower than the LLOQ of OT and was also the lowest LLOQ of all modified peptides. It was thus chosen for further evaluation in human serum.

To determine the LLOQ of [1-(4-bromobutyl)-4-methylpyridin-1-ium]_2_-OT **20** in human serum, a dilution series of peptide **20** was prepared in 50% ACN_aq_/0.1% FA ranging from 1 mM–1 fM. Sample preparation was performed according to Protocol 6′ and samples were analyzed on the API4000 QTRAP. The LLOQ was determined by spiking 100 µL human serum samples with 10 µL peptide **20** standards (1 mM–1 fM). This yielded an LLOQ of 2 nM, representing a 43-fold improvement compared to human serum samples spiked with OT (LLOQ 86 nM).

## Discussion

A MS method that can reliably monitor endogenous peptide hormone levels in human blood would enable a wide range of studies including fundamental (patho) physiological state studies as well as biomarker studies for clinical trials measuring treatment response or identifying patients with abnormal endogenous peptide levels. In the process of developing such a method, we focused on OT as it is one of the most studied neuropeptide/peptide hormone (including hundreds of clinical trials) due to its high relevance in social behavior and reproductive functions and its involvement in disorders such as autisms spectrum disorders, schizophrenia, anxiety and depression (Feifel et al., 2010; Alvares et al., 2017; Ooi et al., 2017). MS has the advantage that it identifies and quantifies analytes by their mass, therefore removing the possibility of detecting metabolites or similar recognition motifs, as it is often the case with immunoassays (McCullough et al., 2013). However, measuring low peptide concentrations in biological samples by MS remains challenging, with several contradicting results regarding the endogenous concentration of OT in blood (Zhang et al., 2011; Brandtzaeg et al., 2016), likely due to uncharacterized matrix effects.

In this work, we highlight the importance of sample preparation for OT analysis and investigated several strategies to improve the quantitation of OT in human serum by MS. This resulted in a robust sample preparation protocol and the introduction of novel derivatization strategies to improve the ionization efficiency of the analyte and thereby the LLOQ towards physiological levels of OT.

Our optimized sample preparation Protocol 6′ (Table 3) displayed good peptide recovery (81–87%), low matrix effects (±16%) and excellent overall method efficiency (71–77%) in human serum OT quantitation using the pre- and post-spike method (50 , 10 and 0.1 µM). Method precision (6.4–7.6%) and accuracy (−7.0–15.7%) were within or close to FDA guidelines (±15%). The optimized sample preparation includes an organic solvent (ice-cold ACN) protein precipitation step, followed by a liquid-liquid extraction with Et_2_O step and solid-phase extraction on an Oasis HLB column (60 mg), before injection into an ESI-MS instrument. The advantage of this protocol lies in its simplicity and ability to provide consistent results over the observed OT concentration range (pM–µM). The protocol was developed on a common MS instrument (API4000 QTRAP) available in many laboratories, and the LLOQ can be pushed further with more sensitive instruments (Supplementary Figure S4), indicating that it is just a matter of time until we will have reached the threshold of reliable quantifying endogenous OT and other peptide hormone levels in blood or other biological samples (e.g., urine, cerebrospinal fluid).

**TABLE 3:** Optimized sample preparation Protocol 6’. RE, MX, and ME for optimized Protocol 6’ were evaluated in human serum *via* the pre- and post-spike method (50 μM, 10 µM, 0.1 µM). Precision and accuracy were determined by spiking samples with 40, 10 and 1.5 µM OT. The OT analogue derivatized with 1-(4-bromobutyl)-4-methylpyridin-1-ium had the lowest LLOQ when evaluated in human serum.

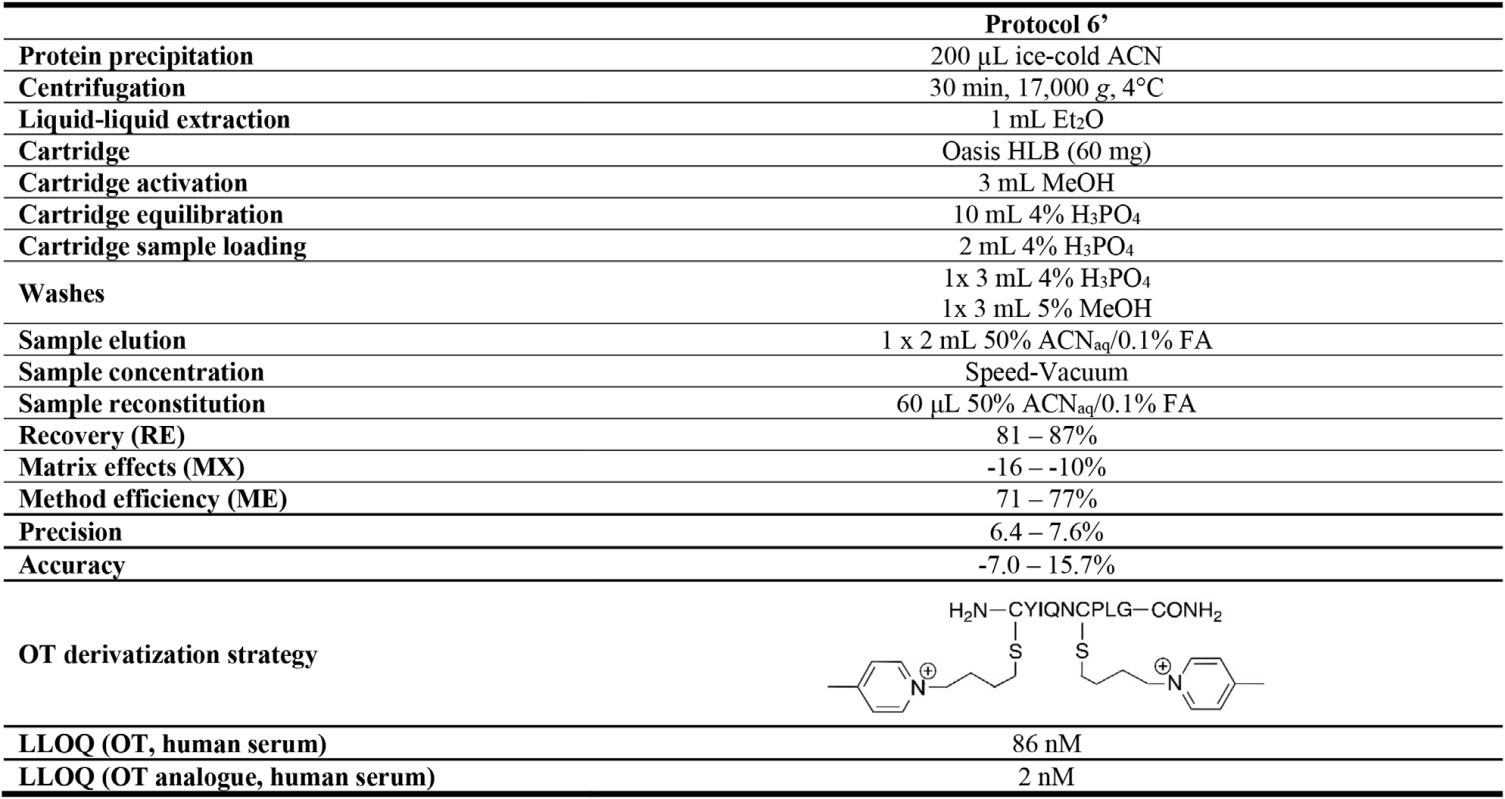

Since the optimized sample preparation method per se was not able to provide a low enough LLOQ for the quantitation of endogenous OT in human serum, we explored an OT derivatization strategy that resulted in another ∼40-fold improvement of the LLOQ from 86 to 2 nM in human serum. The derivatization strategies are based on the hypothesis that sensitivity is affected by the analyte’s ionization efficiency, which is directly linked to its physicochemical properties such as charge and hydrophobicity (Wahl et al., 1993; Page et al., 2007). However, no direct correlation between sensitivity and retention time nor overall charge was observed. However, there was a clear trend between oxidized vs. reduced OT analogues, with oxidized analogues yielding better sensitivity. A likely explanation for this is that the thiol groups of the reduced peptide interact with water molecules through hydrogen bonding leading to the formation of a hydration shell around the peptide. Higher energy is then needed to evaporate the solvent and hydration shell, resulting in poorer ionization and sensitivity.

Given the trajectory of technological development in the MS space, we will continue to see substantial advances pushing the limits of sensitivity and quantitation, as already seen with the more sensitive QTRAP6500. The ability to detect specific masses by MS provides a clear advantage over the commonly used immunoassays that are often prone to specificity issues and false positives. Additionally, MS has a huge potential for further advancements, making it the technique of choice for future bioanalytical studies.

In conclusion, we have achieved the development of an optimized sample preparation protocol and introduced and evaluated new and promising derivatization strategies to improve the sensitivity of detecting OT in biological samples. This provides a solid foundation for a standardized and accessible MS method to quantitate OT levels in biological samples, valuable for OT biomarker studies supporting fundamental research, patient blood screens, treatment monitoring and clinical trials.

## Data Availability

The original contributions presented in the study are included in the article/Supplementary Material, further inquiries can be directed to the corresponding author.

## Abbreviations

ACN, Acetonitrile; HLB, Hydrophobic-lipophilic-balanced; HPLC, High-performance liquid chromatography; LCMS, Liquid chromatography-mass spectrometry; LC-MS/MS, Liquid chromatography-tandem mass spectrometry; LLOQ, Lower limit of quantitation; ME, Method efficiency; MX, Matrix effects; OT, Oxytocin; RE, Recovery.
