# Supplementary material for: Approaches to Improve the Quantitation of Oxytocin in Human Serum by Mass Spectrometry
Source: Front Chem. 2022 Jun 9;10:889154. doi: 10.3389/fchem.2022.889154 (PMC9218718; doi:10.3389/fchem.2022.889154)
Supplement: Supplementary file 1 [file DataSheet1.pdf]

Supplementary Material

Approaches to improve the quantitation of oxytocin in human serum by mass spectrometry.

Anke Hering[1#], Beverly Jieu[1#], Alun Jones[1], Markus Muttenthaler[1,2*]

[1]Institute for Molecular Bioscience, The University of Queensland, Brisbane, St Lucia, QLD 4072, Australia.
[2]Institute of Biological Chemistry, Faculty of Chemistry, University of Vienna, 1090 Vienna, Austria

[*] Correspondence: m.muttenthaler@uq.edu.au

[#] These authors have contributed equally to this work.

1. Materials

All solvents and reagents were used as supplied. Fmoc amino acid derivates (Fmoc-Gly)-OH, (Fmoc-Leu)-OH, (Fmoc-Pro)-OH, (Fmoc-Cys(Trt))-OH, (Fmoc-Asn(Trt))-OH, (Fmoc-Gln(Trt))-O, (Fmoc-Tyr(tBu))-OH (Fmoc-Ile)-OH, (Fmoc-Phe)-OH, (Fmoc-Trp(Boc)-OH, (Fmoc-Arg(Pbf))-OH and (Fmoc-Lys(Boc))-OH were purchased from Iris Biotech (Germany). The rink amide resin (Polystyrene AM RAM; 0.69 mmol/g) was obtained from RAPP Polymere. Triisopropylsilane (TIPS), 1,2-ethanedithiol (EDT), ammonium bicarbonate (NH_4HCO_3), iodine chips, sodium hydroxide, 1,4-dibromobutane, triethylamine, 1-butyl-1H-imidazole, 4-methylpyridine, diisopropylcarbodiimide (DIC), and male human serum (item No. H6914) were obtained from Sigma-Aldrich (USA. The coupling reagent 2-(6-chloro-1-H-benzotriazole-1-yl)-1,1,3,3-tetramethylaminium hexafluorophosphate (HCTU), N,N-diisopropylethylamine (DIPEA), dimethylformamide (DMF) and diethyl ether (Et_2O) from RCI Labscan Limited (Thailand); trifluoroacetic acid (TFA), formic acid (FA), piperidine and methanol (MeOH) from Chem-Supply; dichloromethane (DCM) and acetonitrile (ACN) from Merck (USA). Plastic HPLC inserts were obtained from Agilent Technology (USA, item No. 5182-0549) and glass HPLC inserts from SUPELCO (USA, item No. 24707). Solid-phase extraction cartridges Sep-Pak C_{18} (50 mg) (item No. WAT054955) and Oasis HLB (60 mg) (item No. WAT094226) were bought from Waters (USA). Ethyl(hydroxyimino)cyanoacetate (Oxyma) was purchased from Chem-Impex International (USA).

2. General Procedures

2.1 Solid phase peptide synthesis of OT analogues with hydrophobic and/or charged residues (for investigation of ionisation efficiency)

OT (H-CYIQNCPLG-NH_2) and OT analogues were synthesised on an automated microwave-assisted peptide synthesiser (Liberty Prime, CEM) *via* Fmoc-SPPS on 0.1 mmol scale on a Rink-amide resin (RAM; RAPP Polymer, 0.74 mmol/g). Fmoc deprotection was performed using 25% pyrrolidine in DMF. Amino acid couplings (5 eq.) were carried out with DIC/Oxyma Pure®. Fmoc-AA were activated in a mixture of Fmoc-AA-OH/DIC/Oxyma (1:2:1). Upon completion of the peptide chain, the resin was washed with DCM and MeOH. Cleavage from resin and removal of the side-chain protecting groups was achieved by treatment with TFA/TIPS/EDT/H_2O (92.5: 2.5: 2.5 : 2.5) at 40°C for 40 min. The resin was filtered off, followed by peptide precipitation with ice-cold Et_2O. The peptide suspension was centrifuged, and the peptide pellet was washed 3x with ice-cold Et_2O. The Et_2O was decanted, and the peptide pellet was dissolved in 50% ACN_{aq}/0.1% TFA and lyophilised overnight. The crude peptide was analysed *via* LC-MS. OT was oxidised by stirring the linear peptide overnight in 0.1M ammonium bicarbonate buffer (0.1 mg/mL, pH 8.2). For oxidative folding of OT analogues, the linear peptides (0.1 mg/mL) were dissolved in 20-30% ACN_{aq}/0.1% TFA. An iodine solution (10 mg/mL iodine in methanol) was added dropwise during intensive stirring, until the peptide solution turned slightly yellow/brown; stirring was continued stirring for 1 min. The oxidation was stopped by addition of ascorbic acid until the solution turned colourless. The folded peptides were purified by preparative RP-HPLC (**Table S1**). Cleavage, deprotection, oxidative folding and purification of the peptides was monitored by analytical RP-HPLC and ESI-LC/MS (**Table S1**).

2.2 Quantitation of the peptide concentration to prepare standard solutions

Synthetic peptides contain varying quantities of salts and trapped water molecules from purification/lyophilisation procedures. To determine the actual peptide content and purity, analytical RP-HPLC was performed (**Table S1**) and compared against two peptide standards with known peptide content established by amino acid analysis (OT, vasopressin). Using the Beer-Lambert Law, the peptide concentrations were calculated based on absorbance of standards and samples using calculated extinction coefficients (Buck et al., 1989, Moffatt et al., 2000, Conibear et al., 2012, Kremsmayr and Muttenthaler, 2022).

Table S1: Analytical and preparative HPLC and MS conditions for peptide synthesis and quantitation of peptide concentrations.

**HPLC system**	**Analytical and quantitation:** Shimadzu LC-20AT solvent delivery system, SIL-20AHT auto-injector, SPD-20A Prominence UV/VIS detector (214 nm and 280 nm)<br>**Preparative:** (Waters Delta 600 HPLC System (214 nm)
**Column**	**Analytical:** Agilent-Zorbax C18, 300 Å, 5 µM, 22.1 x 250 mm<br>**Preparative**: Agilent Eclipse C18, 300 Å, 3.5 µM, 2.1 x 50 mm<br>**Quantitation:** Agilent Hypersil Gold C18, 100 x 2.1 mm, 3 µM
**Solvent A**	**Analytical:** H_2O/0.1% FA<br>**Preparative:** H_2O/0.05% TFA<br>**Quantitation:** H_2O/0.05% TFA
**Solvent B**	**Analytical:** ACN/0.1% FA<br>**Preparative:** 90% ACN_{aq}/0.043% TFA<br>**Quantitation:** 90% ACN_{aq}/0.043% TFA
**Flow rate**	**Analytical:** 0.25 mL/min<br>**Preparative:** 16 mL/min<br>**Quantitation:** 0.3 mL/min
**Gradient**	**Analytical:** 0–50% B in 50 min<br>**Preparative:** 0–50% B in 100 min<br>**Quantitation:** 0–90% B in 18 min
**MS system**	**Analytical:** QSTAR Elite, QqTOF<br>**Method development:** API4000 QTRAP, Sciex, TurboIonSpray source

2.3 LC-MS/MS conditions for sample preparation method development

The LC-MS/MS system consisted of a Shimazu HPLC LC-30 AD binary pump system and tandem quadrupole mass spectrometer (API4000 QTRAP, Sciex) with a TurboIonSpray source employed for detection, which was controlled by Analyst software (version 1.6.2). Chromatography was performed as per conditions listed in **Table S2**. The API4000 QTRAP was operated in a positive heated ESI mode using multi reaction monitoring (MRM) transition mode. The MRM transition for OT was optimised for m/z 1007.5 (Q1) → 723.0 (Q3) (**Figure S1**).

All measurements were carried out on a Shimadzu LC-30 HPLC coupled to a API4000 QTRAP, which was operated in positive electrospray ionisation and multiple reaction monitoring (MRM) mass transition mode. The precursor ion (Q1 ion) for OT was *m/z* 1007.5 and the product ion (Q3 ion) was *m/z* 723.0. Fragmentation of Q1 ion broke the peptide bond between Cys^6 and Pro^7, resulting in a **b** (Q3 ion) and **y**-ion (**Figure S1**). The b-ion was used for method development due to its higher signal intensity on the API4000 QTRAP compared to the y-ion.

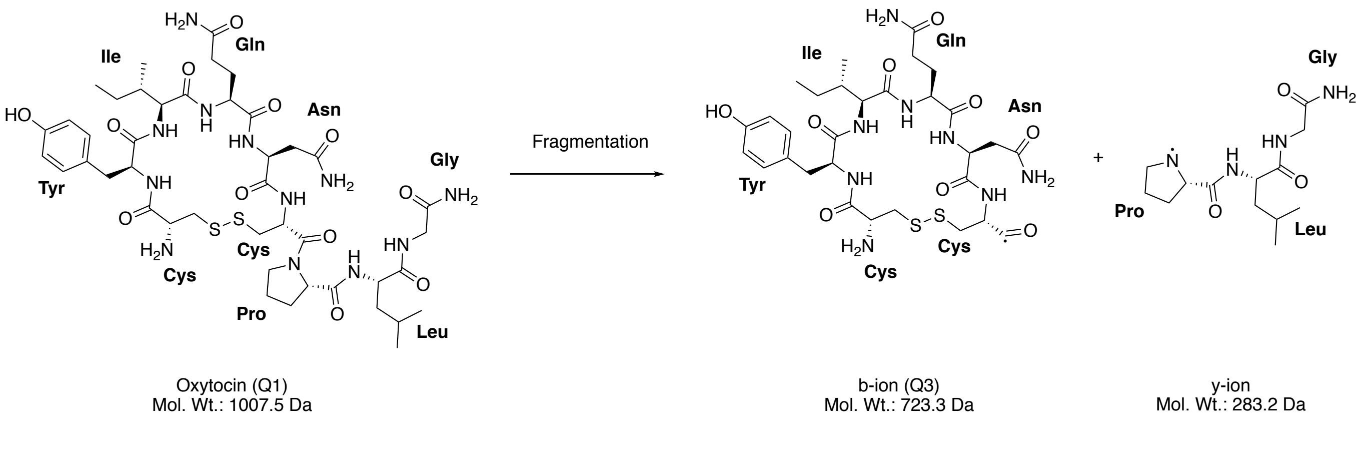

Figure S1: Fragmentation overview of OT in a API4000 QTRAP.

Table S2: LC-MS/MS conditions for analytical method development.

**HPLC system**	Shimazu HPLC LC-30 AD binary pump system
**Column**	Thermo Scientific Hypersil Gold C_{18}, 100 x 2.1 mm, 3.5 µM
**Oven temperature**	45°C
**Solvent A**	H_2O/0.1% FA
**Solvent B**	ACN/0.1% FA
**Flow rate**	0.25 mL/min
**Gradient**	10–90% B in 5 min, 90% B for 3 min, 90–10% B in 0.5 min, 10% B for 6 min
**MS system**	API4000 QTRAP, Sciex, TurboIonSpray source
**Scan type**	MRM
**Entrance potential (V)**	10
**Collision cell exit potential (V)**	15
**Curtain gas (psi)**	30
**Ion spray voltage (V)**	5000
**Ion source temperature (°C)**	500

2.4 Optimisation of MS analysis method

A 0.1 mM solution of the peptide analyte of interest was prepared and directly injected (15 µL/min) into the API4000 QTRAP to optimise the collision energy (CE) and declustering potential (DP). First, a Q1 scan followed by a product ion scan was performed to identify suitable ions for Q1 and Q3. Then the CE and DP were optimised for each Q1/Q3 transition by running a CE (0-130 V) or DP (0-400 V) ramp (**Table S2**).

2.5 Evaluation of peptide adsorption to glass or plastic HPLC inserts

OT standard solution of 10 µM in H_2O, 25%, 50% and 75% ACN_{aq}/0.1% FA were prepared and added to the plastic HPLC inserts (Agilent Technology, item No. 5182-0549) and glass HPLC inserts (SUPELCO, item No. 24707). Each standard was analysed every hour for 24 hours by injecting 1 µL into the API4000 QTRAP. Three independent experiments were carried out. The LC-MS/MS peaks were integrated using Analyst software (version 1.6.2) and all results were expressed as a percentage relative to the peak area of OT in 50% ACN_{aq}/0.1% FA in plastic inserts.

2.6 Evaluation of injection volume

A 10 µM OT standard solution in 50% ACN_{aq}/0.1% FA in plastic HPLC inserts was prepared. 1, 5, 10 and 20 µL of OT standard solution were injected (n = 3) into the API4000 QTRAP. Five independent experiments were carried out. LC-MS/MS peaks were integrated using Analyst software (version 1.6.2).

2.7 Calibration curve and determining the LLOQ of mass spectrometers

Dilution series of 14 OT standards ranging from 1 mM to 0.1 fM in 50% ACN_{aq}/0.1% FA were prepared and 10 µL of each standard solution injected to the LC-MS/MS instrument for analysis. Standard curves were prepared in three independent experiments and analysed in triplicates. LC-MS/MS peaks were integrated using Analyst software (version 1.6.2). For the standard curve, the logarithmic (log_{10}) peak areas were plotted against the logarithmic (log_{10}) OT concentrations. The linear range was determined by the maximum number of points that could be included for the R^2 coefficient to remain ≥0.9. LLOQ was determined by visual evaluation of the standard curve (smallest value on the linear range, before the plateau) and had to be ≥ 5x of the blank signal). HPLC conditions: 0-90% B in 3 min at 0.25 mL/min (A: H_2O/0.1% FA; B: ACN/0.1% FA). HPLC column: Agilent Technologies, Hypersil Gold C_{18}, 100 x 2.1 mm, 3 µm. MS settings were chosen according to tuning results (**Table S2**).

2.8 Evaluation of sample preparation protocols using matrix effect, peptide recovery and overall method efficiency

Six sample preparation protocols were evaluated towards matrix effect (MX), peptide recovery (RE) and overall method efficiency (ME). 100 µL human serum samples were spiked with 10 µL OT standard solutions at three concentrations (0.1 µM, 10 µM and 50 µM), yielding final OT serum concentrations of 4.5 µM, 0.91 µM and 9.09 nM, respectively. Protocols 1 to 3 were performed on a Sep-Pak (50 mg) cartridge (item No. WAT054955) from Waters and on an Oasis HLB (60 mg) cartridge (item No. WAT094226) from Waters, while Protocols 4, 5 and 6 were only tested on Oasis HLB cartridges. MX, RE and ME were determined *via* the quantitative pre- and post-spike method (Matuszewski et al., 2003). Each protocol was performed on three samples, and each sample was analysed in triplicates. HPLC conditions: 0–90% B in 3 min at 0.25 mL/min (A: H_2O/0.1% FA; B: ACN/0.1% FA). HPLC column: Agilent Technologies, Hypersil Gold C_{18}, 100 x 2.1 mm, 3 µM. MS settings were chosen according to tuning results (**Table S2**).

2.9 Evaluation of reconstitution volume and LLOQ of sample preparation Protocol 6'

To determine the MX, RE and ME of each reconstitution volume, 100 µL human serum samples were spiked with 10 µL of OT standard solution (50 µM) and sampled according to Protocol 6' (**Table 3**). Reconstitution volumes of 40, 60, 80 or 100 µL of 50% ACN_{aq}/0.1% FA were analysed. MX, RE and ME were determined *via* the quantitative pre- and post-spike method. 10 µL of each reconstituted sample was analysed *via* LC-MS/MS and the chromatographic peaks were integrated using Analyst software (version 1.6.2). To determine the effect of reconstitution volume on LLOQ, 100 µL of human serum samples were spiked with 10 µL of OT standard solutions ranging from 0.1 pM to 1 mM, sampled according to Protocol 6' and reconstituted in 40, 60, 80 or 100 µL of 50% ACN_{aq}/0.1% FA. 10 µL of each sample was analysed by the API4000 QTRAP. The LLOQ of each reconstitution volume was calculated as per Section 2.7.

2.10 Evaluation of intraday accuracy and precision of Protocol 6'

A dilution series of 11 OT standards (0.1 pM to 1 mM) were prepared and analysed by the API4000 QTRAP to produce an OT standard curve (n=5). This standard curve was used to calculate the concentration of OT samples prepared with Protocol 6'. 100 µL human serum samples were spiked with 10 µL OT standards (final human serum concentration: 1.5 µM, 10 µM and 40 µM) and purified with Protocol 6'. Samples were reconstituted with 60 µL 50% ACN_{aq}/0.1% FA, and 10 µL of each reconstituted sample was analysed *via* LC-MS/MS. Chromatographic peaks were integrated using Analyst software (version 1.6.2). Precision was expressed as a percentage of the standard deviation from the mean value of the five experiments, and accuracy as a percentage of the error from the expected value (Administration, 2018).

2.11 Evaluation of LLOQ of OT across different mass spectrometers

A dilution series of OT ranging from 1 fM to 0.1 mM in 50 % ACN, 0.1 % FA was analysed on different mass spectrometers, including four quadrupole time-of-flight (QTOF) mass spectrometers (TripleTOF 6600, TripleTOF 5600, X500R, QstarElite) and two triple quadrupole instruments (API4000 QTRAP, QTRAP6500) (**Figure S4**). All mass spectrometers were operated in positive ESI-mode. The sensitivity of each instrument was determined by calculating the LLOQ of OT as per Section 2.7.

2.12 Evaluation of OT analogue hydrophobicity

The purity and relative hydrophobicity of the synthesised peptides was analysed *via* UV at 214 nm on a RP-HPLC system (column: Hypersil Gold, 100 x 2.1 mm, 3 µM; method: 0-50 % solvent B in 50 min, 0.3 mL/min). 10 µL of the peptide stock solution (3 mg/mL in 50% ACN, 0.1 % FA) were injected. The data were collected and processed using Shimadzu-Lab solution software (version 5.97). The retention time of each peptide was normalised to OT by subtracting OT`s retention time from the peptide's retention time.

2.13 Synthesis of 4-bromo-N,N,N-triethylethan-1-aminium (i)

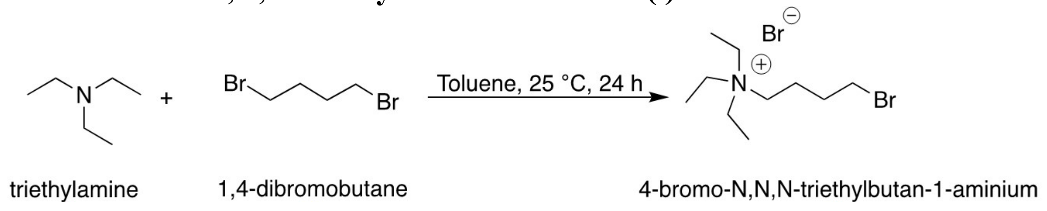

1 eq of triethylamine (700 µL) was added dropwise to a solution of 10 eq of 1,4-dibromobutane (6 mL) in 5 mL anhydrous toluene under inert atmosphere and stirred at room temperature (25ºC) for 24 h. The solvent was evaporated, and the remaining oil was washed 3x with Et_2O. The oil was dried in a desiccator, and 500 mg final product was obtained (32% yield) which was analysed by NMR and MS. 1H NMR (400 MHz, D_2O) δ 1.29 (t, 9H, J = 7.4 Hz, H6, H7 and H8) 1.87 (dt, 2H, J = 14.0, 6.9 Hz, H9), 1.95 (q, 2H, J = 6.6, 6.6, 6.2, 4.5 Hz, H10), 3.21 (h, 2H, J = 5.9, 4.1, 3.5, 3.1 Hz, H2), 3.31 (q, 6H, J = 7.3 Hz, H3, H4 and H5), 3.57 (t, 2H, J = 6.2 Hz, H11). Molecular weight = 237.2 g/mol. NMR spectrum can be found in Section 3.4.

2.14 Synthesis of 3-(4-bromobutyl)-1-butyl-1H-imidazol-3-ium (ii)

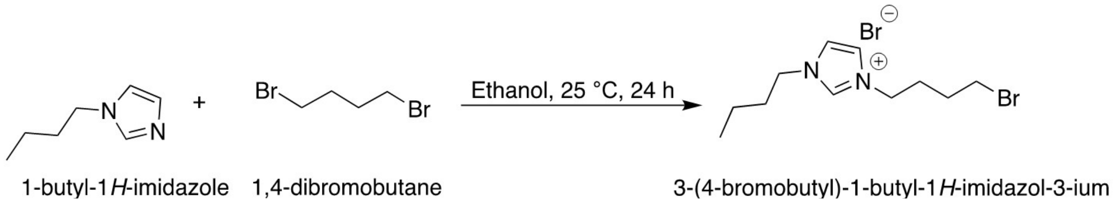

1 eq of 1-butyl-1H-imidazole (650 µL) was added dropwise to a solution of 10 eq of 1,4-dibromobutane (6 mL) in 5 mL anhydrous ethanol under inert atmosphere and stirred at room temperature (25ºC) for 24 h. The solvent was then evaporated, and the remaining oil was washed 3x with Et_2O. The oil was dried in a desiccator, and 180 mg final product was obtained (96% yield) which was analysed by NMR and MS. 1H NMR (400 MHz, D_2O) δ 0.93 (t, 3H, J = 7.4 Hz, H9), 1.32 (h, 2H, J = 7.4 Hz, H8), 1.88 (dq, 4H, J = 14.6, 7.3, 6.8 Hz, H7, H12), 2.06 (p, 2H, J = 7.2 Hz, H11), 3.53 (t, 2H, J = 6.4 Hz, H13), 4.22 (t, 2H, J = 7.1 Hz, H6), 4.26 (t, 2H, J = 7.1 Hz, H10), 7.53 (d, 2H, J = 2.1 Hz, H1, H2). Molecular weight = 260.6 g/mol. NMR spectrum can be found in Section 3.4.

2.15 Synthesis of 1-(4-bromobutyl)-4-methylpyridin-1-ium (iv)

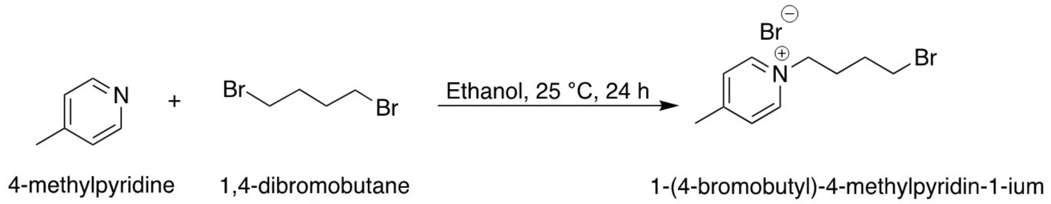

1 eq of 4-methylpyridine (500 µL) was added dropwise to a solution of 10 eq of 1,4-dibromobutane (6 mL) in 5 mL anhydrous ethanol under inert atmosphere and stirred at room temperature (25ºC) for 24 h. The solvent was evaporated, and the remaining oil was washed 3x with Et_2O. The oil was dried in desiccator and 150 mg final product was obtained (100% yield) which was analysed by NMR and MS. 1H NMR (400 MHz, D_2O) δ 1.93 (p 2H, J = 8.5, 6.9, 6.4, 6.4 Hz, H10), 2.17 (p, 2H, J = 7.6 Hz, H9), 2.66 (s, 4H, H7), 3.54 (t, 2H, J = 6.4 Hz, H11), 4.59 (t, 2H, J = 7.4 Hz, H8), 7.89 (d, 2H, J = 6.4 Hz, H1, H3), 8.67 (d, 2H, J = 6.8 Hz, H4, H6). Molecular weight = 229.1 g/mol. NMR spectrum can be found in Section 3.4.

2.16 Alkylation of the thiol groups of reduced OT

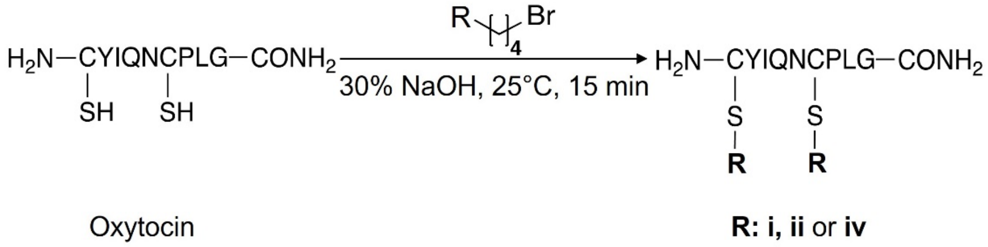

Reduced OT and 2 eq. of ligands **i**, **ii**, **iii** and **iv** were mixed in 30% aqueous NaOH solution and incubated at 25°C for 15 min. The reaction was stopped by adjusting the pH to 3 with TFA. The reaction mixture was diluted with H_2O/0.05 % TFA and purified with preparative RP-HPLC using a linear gradient 0–50% B (solvent A: H_2O/0.05% TFA; B: 90% ACN_{aq}/0.043% TFA) in 100 min at 16 mL/min while monitoring UV absorbance at 214 nm.

3. Results

3.1 Method development

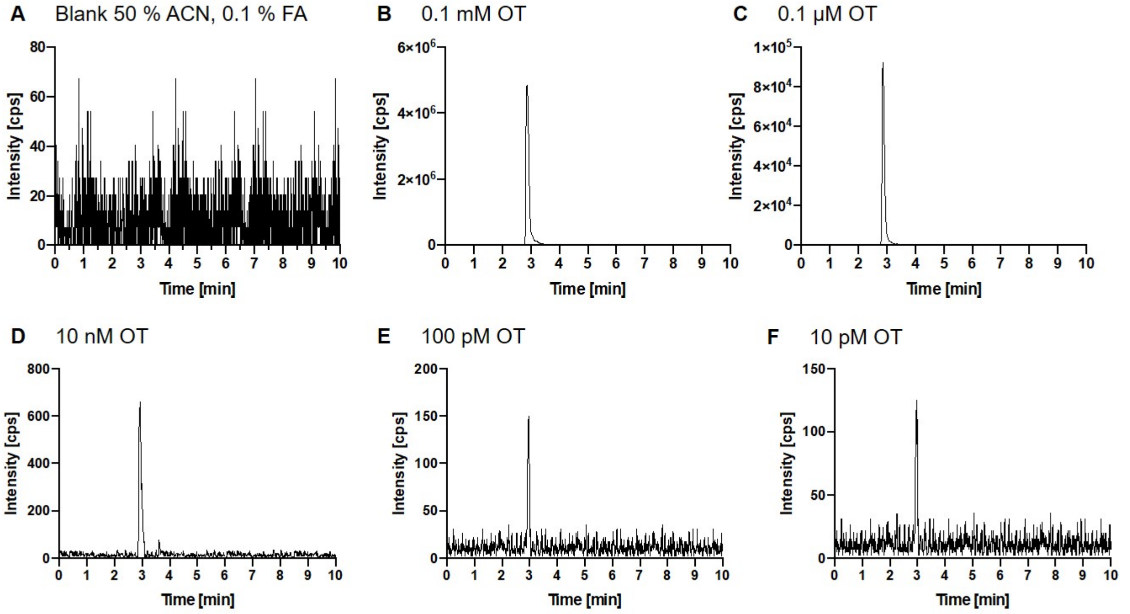

Figure S2: Distinction of OT standard injections from blank signal to determine LLOQ of the API4000 QTRAP. The LLOQ is at least ≥ 5x of the blank signal**.** (**A**) 10 µL injection of blank: 50% ACN_{aq}/0.1% FA. (**B**) 10 µL injection of a 0.1 mM OT standard. (**C**) 10 µL injection of a 1 µM OT standard. (**D**) 10 µL injection of a 10 nM OT standard. (**E**) 10 µL injection of a 100 pM OT standard (**F**) 10 µL injection of a 10 pM OT standard. HPLC column: Agilent, Hypersil Gold C_{18}, 2.1x100 mm, 3.5 µM. HPLC method: 0–90% B in 5 min, 0.25 mL/min. Solvent A: H_2O/0.1% FA. Solvent B: ACN/0.1% FA.

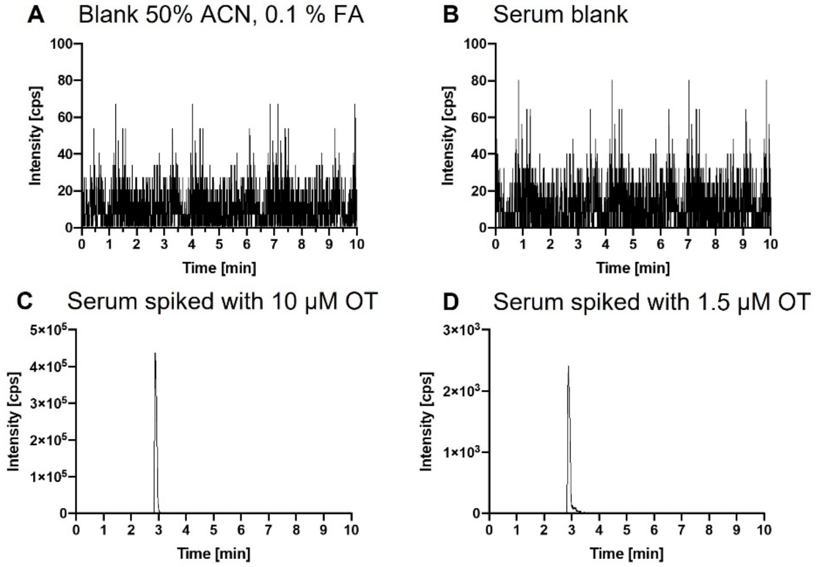

Figure S3: Distinction of OT purified with Protocol 6' from blank signal to determine LLOQ. The LLOQ is at least ≥ 5x of the blank signal**.** (**A**) Blank: 50% ACN_{aq}/0.1% FA. (**B**) Non-spiked human serum blank purified with Protocol 6'. (**C**) Human serum spiked with 10 µM OT. (**D**) Human serum spiked with 1.5 µM OT. HPLC column: Agilent, Hypersil Gold C_{18}, 2.1x100 mm, 3.5 µM. HPLC method: 0–90% B in 5 min, 0.25 mL/min. Solvent A: H_2O/0.1% FA. Solvent B: ACN/0.1% FA.

Table S3: Recovery, matrix effect and method efficiency of sample preparation protocols. Recovery (RE), matrix effect (MX) and method efficiency (ME) were determined *via* the pre-and post-spike method by spiking 100 µL serum samples or extracted serum samples with 10 µL OT standard (0.1 µM, 10 µM, 50 µM) to give final OT serum concentrations of 4.5 µM, 0.91 µM and 9.09 nM. MX, RE and ME are expressed as mean values ± SD of four independent experiments. Samples were analysed on an API4000 QTRAP. MX values within -30–30% are highlighted in green, between ±30–60% in yellow, and outside of ±60% in red. RE values between 80–105% are highlighted in green, 30–80% or 105–120% in yellow, and <30% or >120% in red. ME values between 80–105% are highlighted in green, 30–80% in yellow, and 0–30% in red.

	Sep-Pak (50 mg)								
OT serum conc.	**Protocol 1**			**Protocol 2**			**Protocol 3**		
	MX (%)	RE (%)	ME (%)	MX (%)	RE (%)	ME (%)	MX (%)	RE (%)	ME (%)
4.5 µM	-85 ± 26	248 ± 6	0	-85 ± 10	189 ± 6	0	-60 ± 1	155 ± 1	0
0.91 µM	-94 ± 5	308 ± 12	0	-67 ± 12	188 ± 16	0	-6 ± 15	107 ± 13	101 ± 23
9.09 nM	-94 ± 10	520 ± 39	0	-41 ± 11	223 ± 12	0	-40 ± 10	120 ± 22	0

	Oasis HLB (60 mg)								
OT serum conc.	**Protocol 1**			**Protocol 2**			**Protocol 3**		
	MX (%)	RE (%)	ME (%)	MX (%)	RE (%)	ME (%)	MX (%)	RE (%)	ME (%)
4.5 µM	-14 ± 3	94 ± 7	81 ± 9	-17 ± 38	120 ± 20	0	-76 ± 11	181 ± 11	0
0.91 µM	-93.7 ± 2	138 ± 2	0	-91 ± 14	884 ± 14	0	-86 ± 4	275 ± 53	0
9.09 nM	-8 ± 18	117 ± 18	0	-74 ± 12	217 ± 20	0	-74 ± 8	345 ± 32	0

	Oasis HLB (60 mg)								
OT serum conc.	**Protocol 4**			**Protocol 5**			**Protocol 6**		
	MX (%)	RE (%)	ME (%)	MX (%)	RE (%)	ME (%)	MX (%)	RE (%)	ME (%)
4.5 µM	+10 ± 16	3 ± 33	3 ± 3	+1 ± 6	39 ± 3	39 ± 18	+6 ± 3	87 ± 8	82 ± 8
0.91 µM	-10 ± 19	9 ± 56	8 ± 5	-29 ± 10	84 ± 29	60 ± 21	+10 ± 11	88 ± 9	80 ± 14
9.09 nM	-33 ± 41	47 ± 85	31 ± 19	-15 ± 11	110 ± 15	94 ± 22	+7 ± 14	102 ± 1	95 ± 12

Table S4: Effect of reconstitution volume on matrix effect, recovery, method efficiency. 100 µL serum samples were spiked with 10 µL of 50 µM OT before and after sample preparation with Protocol 6.

**Volume**	**MX (%)**	**RE (%)**	**ME (%)**	**LLOQ (nM)**
40 µL	+51 ± 9	+115 ± 8	56 ± 2	243 ± 87
60 µL	-16 ± 3	-85 ± 4	71 ± 6	86 ± 17
80 µL	+188 ± 56	52 ± 10	0	86 ± 25
100 µL	+123 ± 20	75 ± 11	0	93 ± 37
200 µL	+6 ± 3	87 ± 8	82 ± 8	162 ± 24

Table S5: Matrix effect, recovery, and method efficiency of Protocol 6’. Human serum was spiked with OT to give final OT concentrations of 1.5 µM, 10 µM and 40 µM. The spiked human serum samples were purified with Protocol 6’ and analysed.

**Concentration**	**MX (%)**	**RE (%)**	**ME (%)**
1.5 µM	-16 ± 3	85 ± 4	71 ± 6
10 µM	-10 ± 8	81 ± 6	73 ± 7
40 µM	-12 ± 5	87 ± 10	77 ± 8

Table S6: Intraday precision and accuracy. Intraday precision and accuracy of the LC-MS/MS assay to quantify OT in human serum at three OT concentrations measured in five independent experiments.

Theoretical OT concentration	Calculated OT concentration from standard curve	Observed OT concentration in human serum	Precision	Accuracy
1.5 µM	1.4 µM	1.68 ± 0.11 µM	6.6 %	15.7 %
10 µM	11.2 µM	10.14 ± 0.76 µM	7.6 %	-6.7 %
40 µM	41.4 µM	38.72 ± 2.48 µM	6.4 %	-7.0 %

Table S7: Conversion of standard concentrations. The molecular weight of OT is 1007.19 g/mol. For simplicity, 1000 g/mol was used for conversions.

Concentration			
g/mL		Molarity	
1	mg/mL	1	mM
100	µg/mL	100	µM
10	µg/mL	10	µM
1	µg/mL	1	µM
100	ng/mL	100	nM
10	ng/mL	10	nM
1	ng/mL	1	nM
100	pg/mL	100	pM
10	pg/mL	10	pM
1	pg/mL	1	pM
100	fg/mL	100	fM
10	fg/mL	10	fM
1	fg/mL	1	fM

3.2 Improving sensitivity

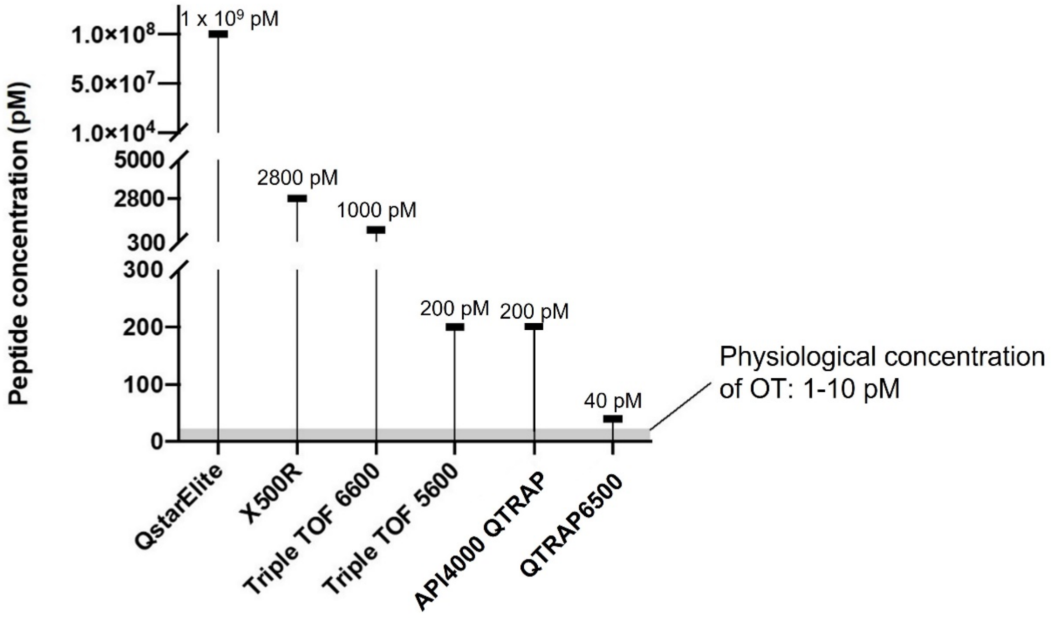

Figure S4: Comparison of LLOQs of OT across different mass spectrometers. A dilution series of OT ranging from 0.1 mM to 1 fM in 50% ACN_{aq}/0.1% FA was measured (n=3) on different MS instruments.

Table S8: Calculated and observed molecular weight and tuning parameters of OT and OT analogues (API4000 QTRAP). DP: Declustering potential, CE: Collision energy, * C-terminal amide.

Peptide ID	Sequence	Molecular weight (Da)	Observed molecular weight on LCMS [M+1]	Q1/Q3	DP	CE
**Reduced**						
1*	CYIQNCPLG*	1008.4521	1009.6723	1009/992	50	36
2*	WCYIQNCPLG*	1194.5314	1193.4988	598/590	80	17
3*	RCYIQNCPLG*	1164.5532	583.2662 [M+2]	583/575	95	19
4*	FCYIQNCPLG*	1155.5205	578.7556 [M+2]	579/388	80	16
5*	KCYIQNCPLG*	1136.5470	569.2714 [M+2]	569/285	90	24
6*	RWCYIQNCPLG*	1350.6325	676.3097 [M+2]	676/1069	110	27
7*	RKCYIQNCPLG*	1292.6481	647.3193[M+2]	647/285	130	34
8*	RFCYIQNCPLG*	1311.6216	656.5362 [M+2]	656/285	60	26
9*	WFCYIQNCPLG*	1341.5998	671.7955 [M+2]	672/630	90	20
10*	KFCYIQNCPLG*	1283.6154	642.8551 [M+2]	643/285	95	28
11*	RWKCYIQNCPLG*	1478.7274	740.3539 [M+2], 493.9093 [M+3]	494/704	43	16
12*	RWFCYIQNCPLG*	1497.7009	749.8463 [M+2]	750/285	110	32
13*	WFKCYIQNCPLG*	1469.6947	735.5387 [M+2]	736/1187	60	24
14*	RWKFCYIQNCPLG*	1625.7958	814.3926 [M+2], 542.9295 [M+3]	578/570	100	16
15*	WKCYIQNCPLG*	1323.5980	662.5375 [M+2]	662/285	60	32
16*	RFKCYIQNCPLG*	1440.7500	1440.6578, 720.5478 [M+2]	721/285	52	33
17*	C(i)YIQNC(i)PLG*	1321.7939	660.7589 [M+2]	661/205	80	50
18*	C(ii)YIQNC(ii)PLG*	1367.7819	683.7989 [M+2]	684/578	70	33
19*	C(iii)YIQNC(iii)PLG*	1123.3130	662.5395 [M+2]	480/683	90	18
20*	C(iv)YIQNC(iv)PLG*	1305.6659	652.6889 [M+2]	652/562	60	27

**Oxidised**						
1	CYIQNCPLG*	1006.4521	1007.3429	1007/723	88	39
2	WCYIQNCPLG*	1192.5314	1193.4988, 597.2834 [M+2]	597/583	60	24
3	RCYIQNCPLG*	1162.5532	582.2603 [M+2]	582/285	105	25
4	FCYIQNCPLG*	1153.5205	577.7535 [M+2]	578/570	100	16
5	KCYIQNCPLG*	1134.5470	568.4462 [M+2]	568/285	110	26
6	RWCYIQNCPLG*	1348.6325	675.6786 [M+2]	676/285	100	28
7	RKCYIQNCPLG*	1290.6481	646.3077 [M+2], 431.2118 [M+3]	431/610	71	15
8	RFCYIQNCPLG*	1309.6216	655.7994 [M+2]	656/285	100	25
9	WFCYIQNCPLG*	1339.5998	1340.5895, 670.8161 [M+2]	671/285	90	25
10	KFCYIQNCPLG*	1281.6154	641.7989 [M+2]	642/500	115	28
11	RWKCYIQNCPLG*	1476.7274	1477.7120, 739.3597 [M+2]	493/703	70	15
12	RWFCYIQNCPLG*	1495.7009	1496.6790, 748.8648 [M+2]	749/285	62	32
13	WFKCYIQNCPLG*	1467.6947	734.5238 [M+2]	735/285	60	36
14	RWKFCYIQNCPLG*	1623.7958	1624.7633, 812.8977 [M+2]	542/776	58	19
15	WKCYIQNCPLG*	1321.5980	662.5375 [M+2]	662/285	60	35
16	RFKCYIQNCPLG*	1438.7500	1438.9775, 719.8518 [M+2]	721/285	52	33

3.3 OT analogues – HPLC traces and standard curves.

Conversion of peptide concentrations are as per **Table S8**. * denotes the peptide in its reduced form.

Peptide 1*

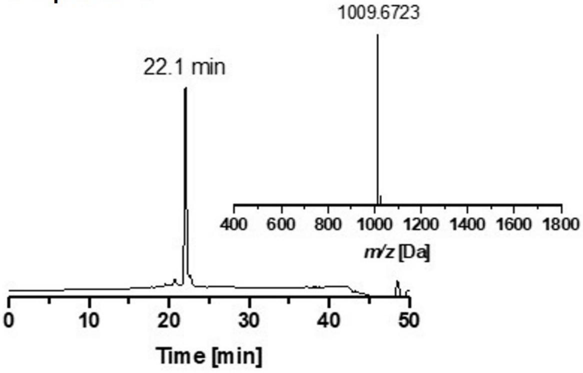

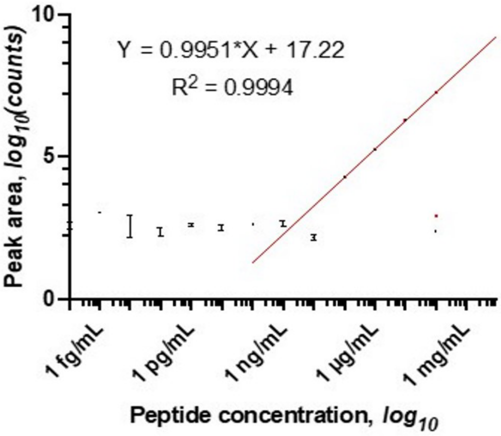

Peptide 1

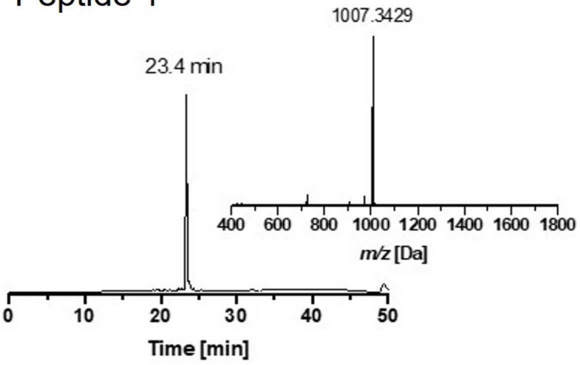

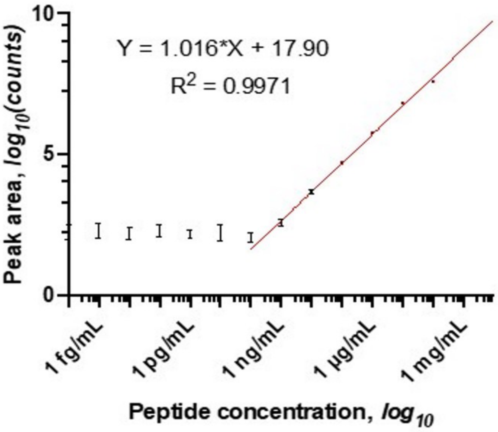

Peptide 2*

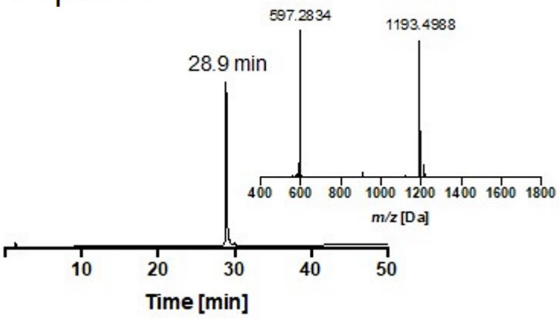

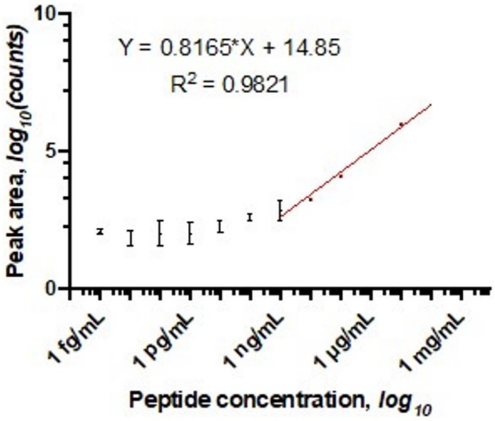

Peptide 2

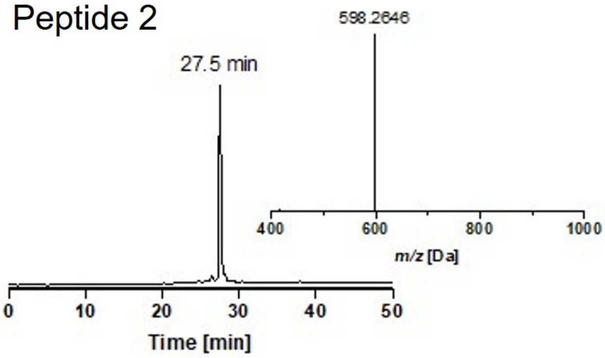

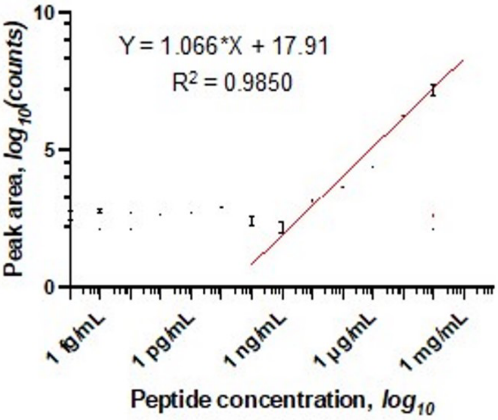

Peptide 3*

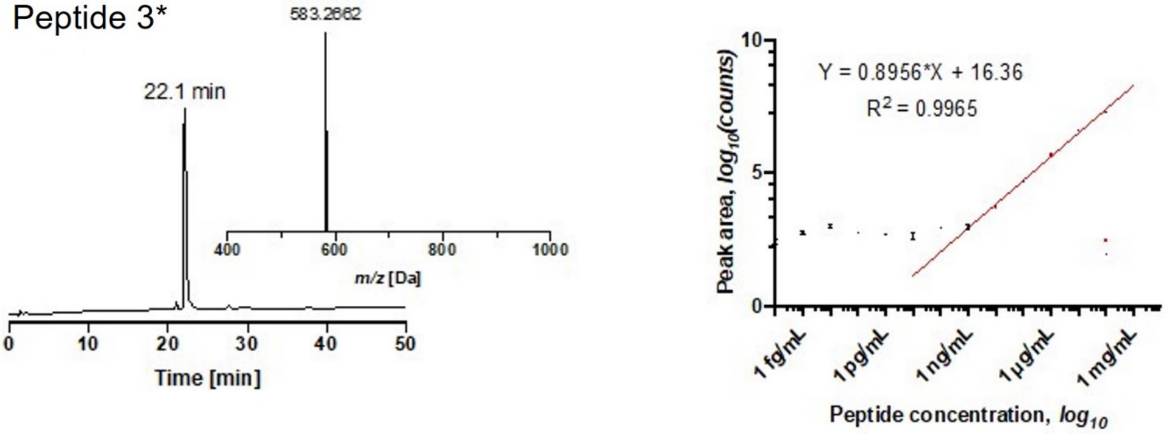

Peptide 3

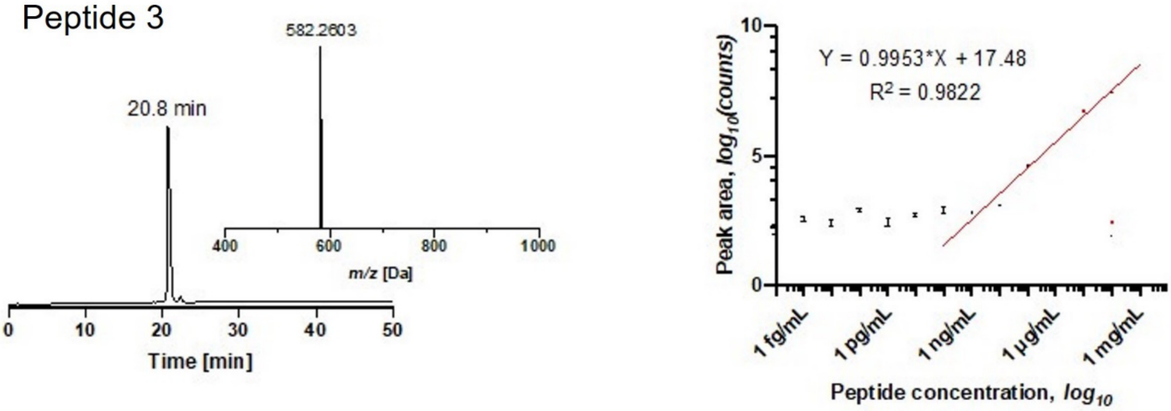

Peptide 4*

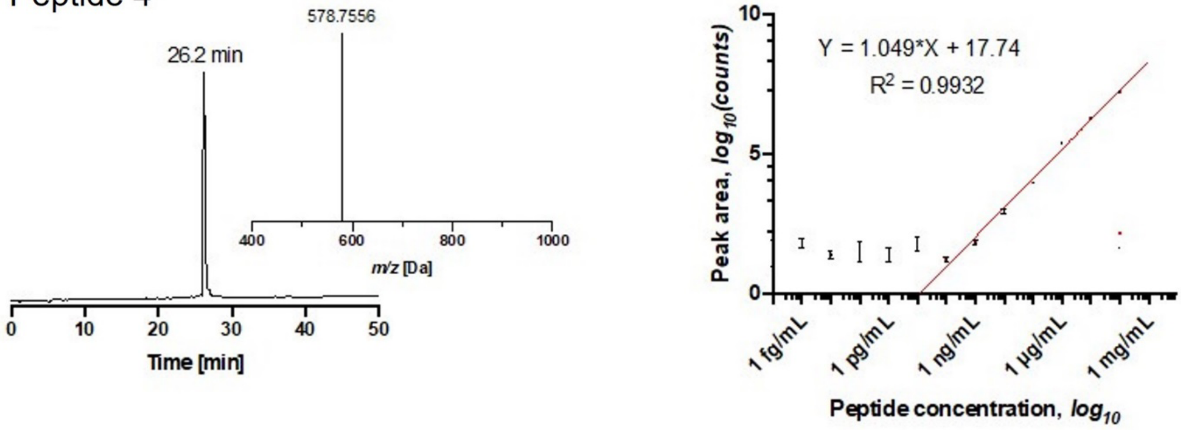

Peptide 4

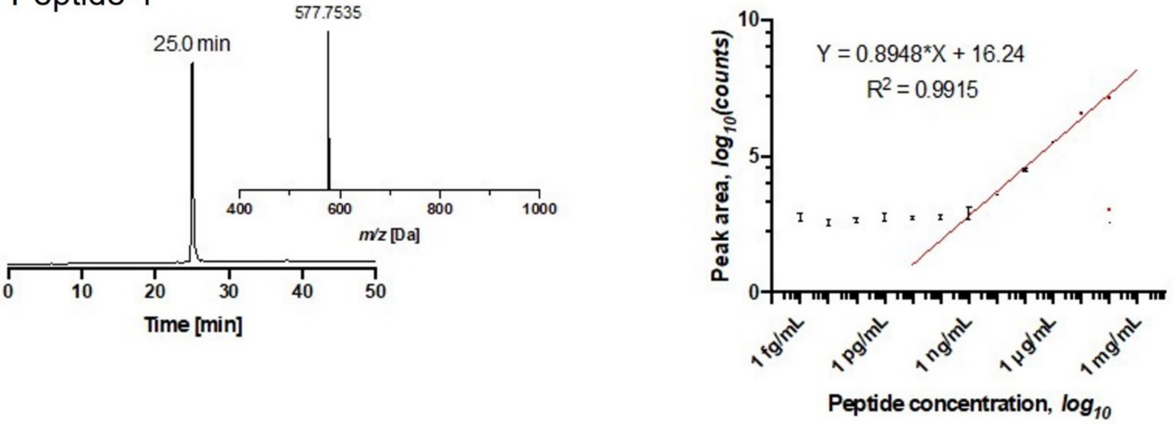

Peptide 5*

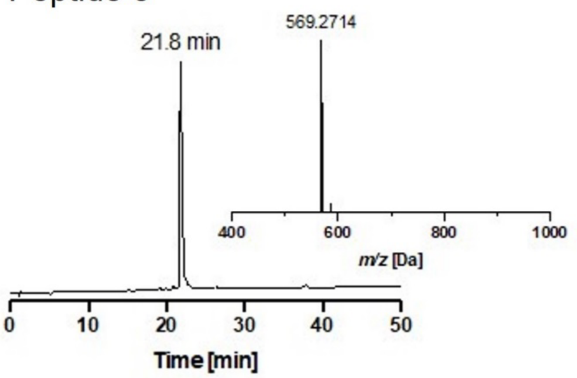

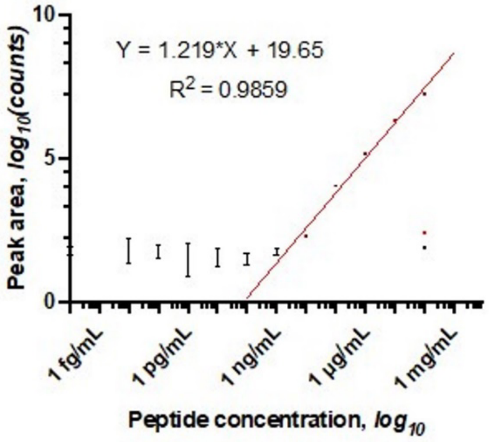

Peptide 5

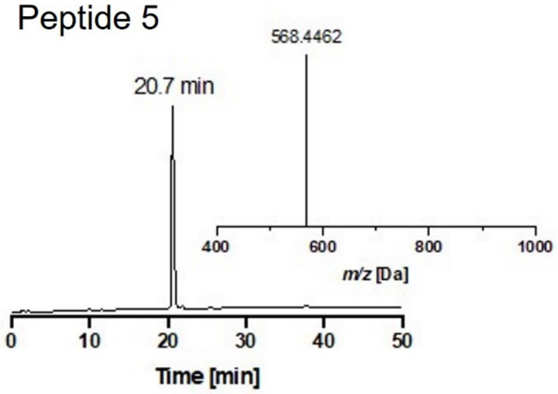

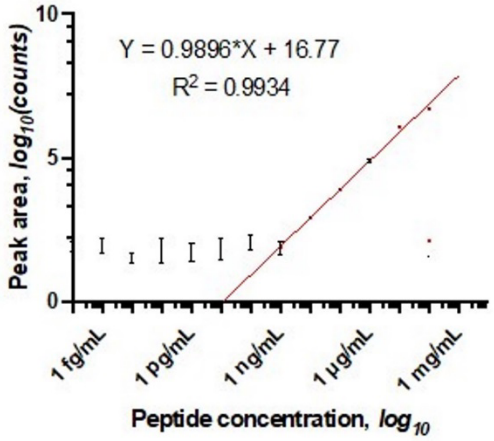

Peptide 6*

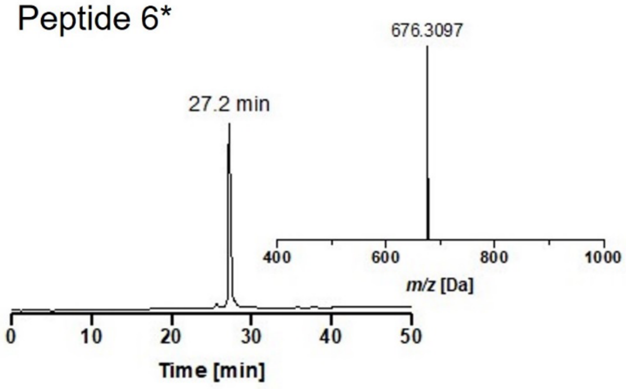

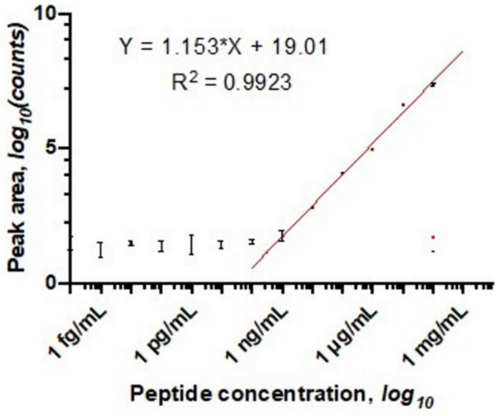

Peptide 6

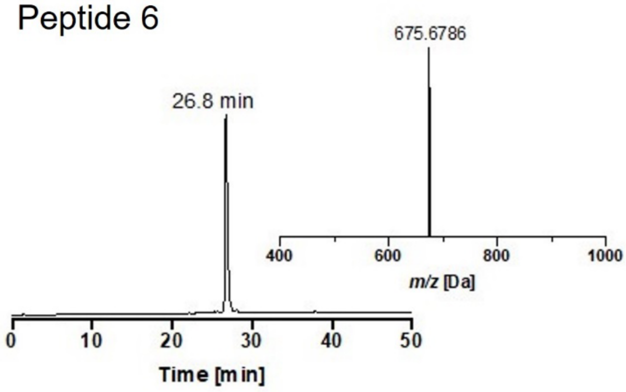

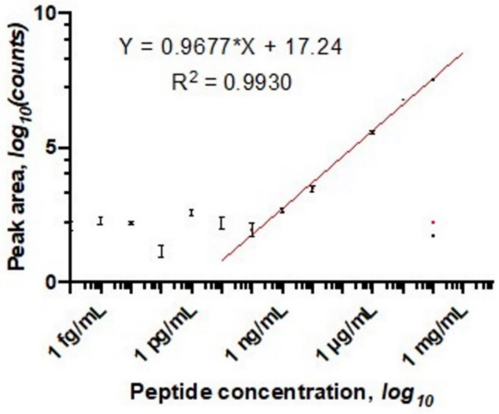

Peptide 7*

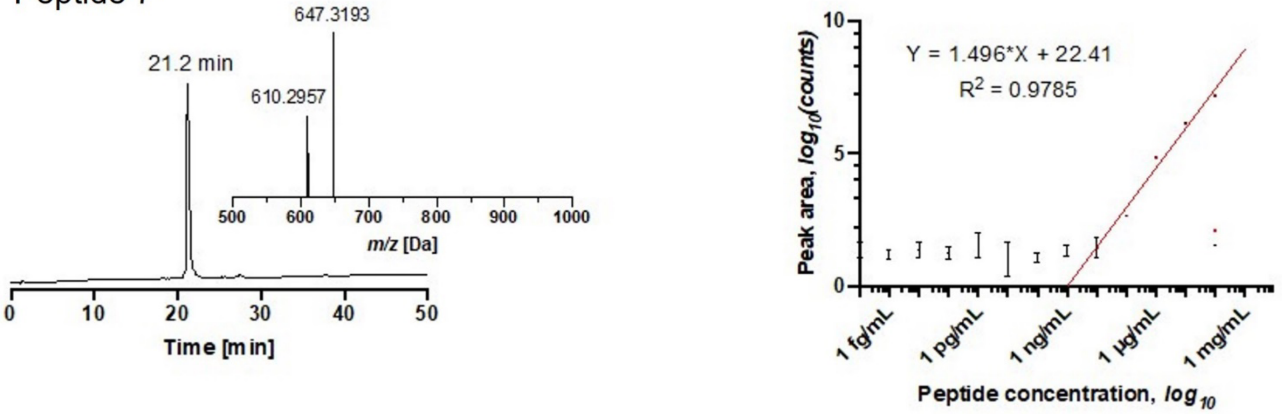

Peptide 7

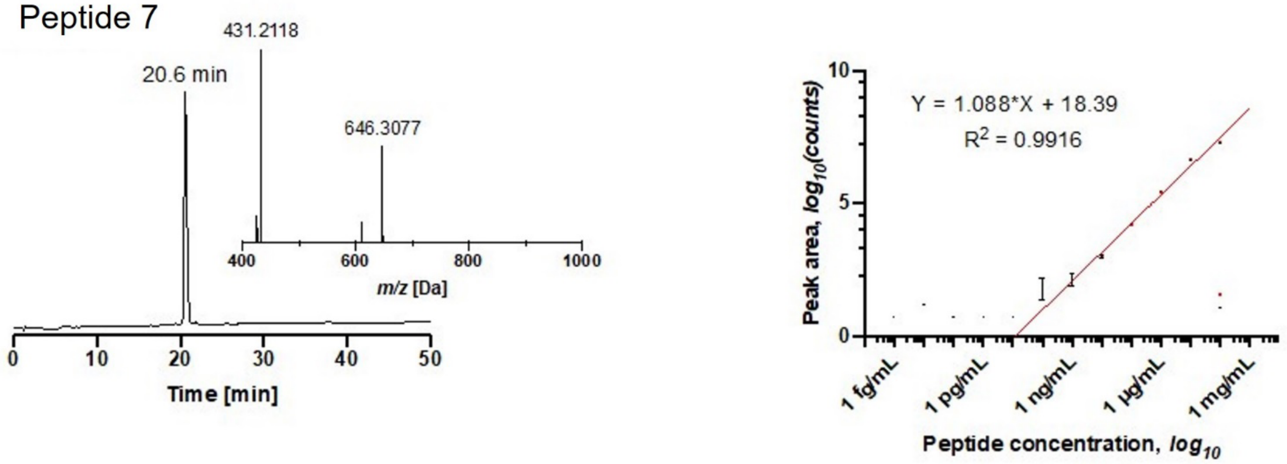

Peptide 8*

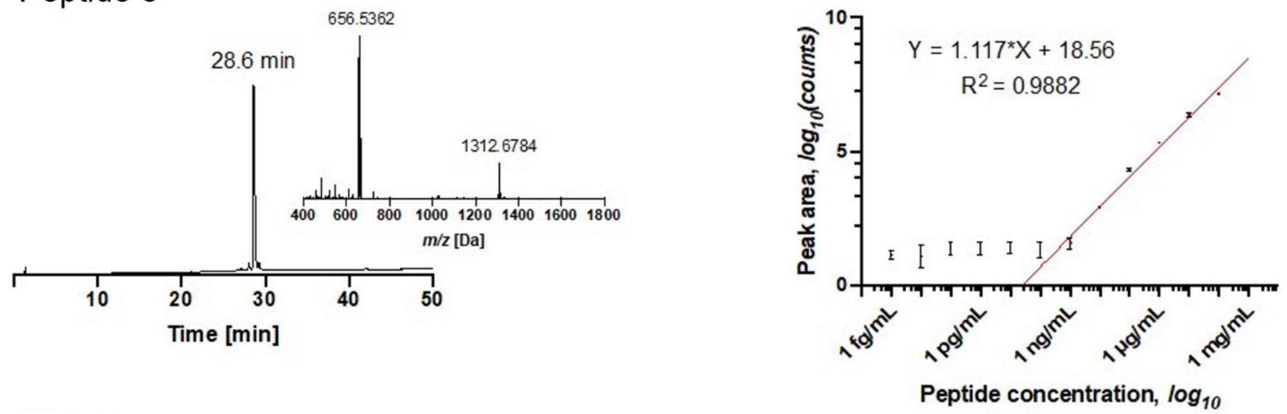

Peptide 8

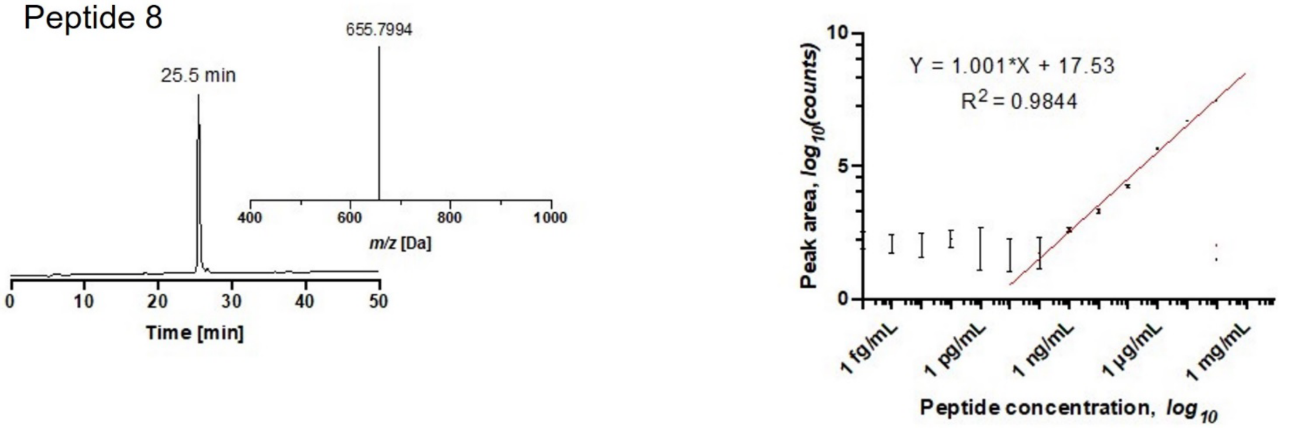

Peptide 9*

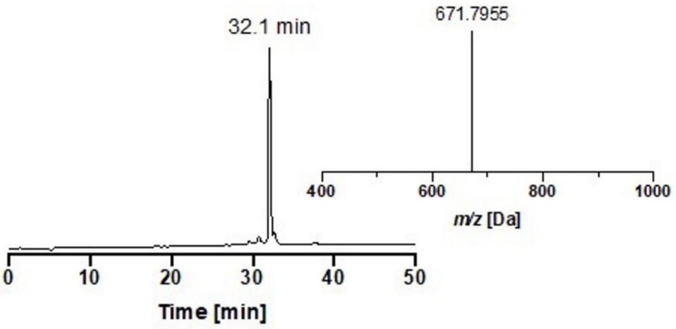

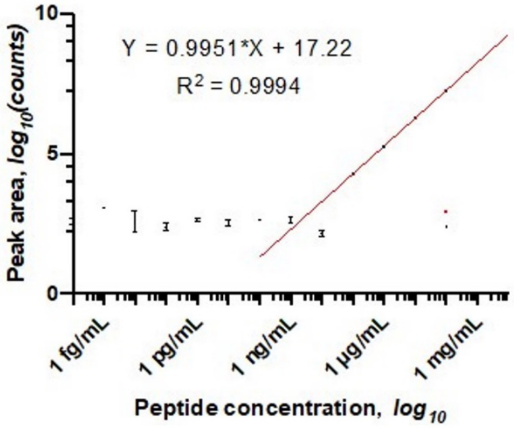

Peptide 9

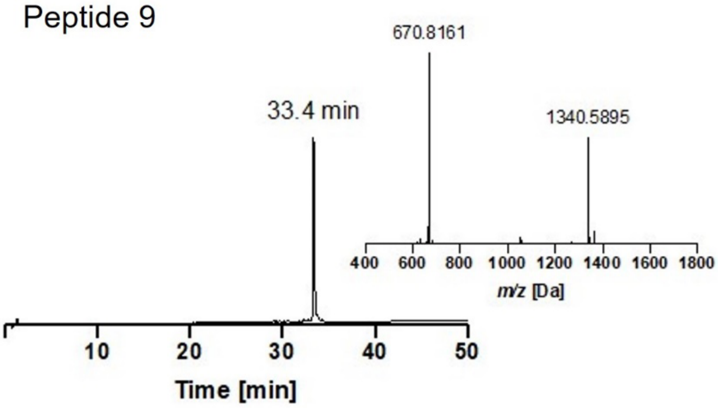

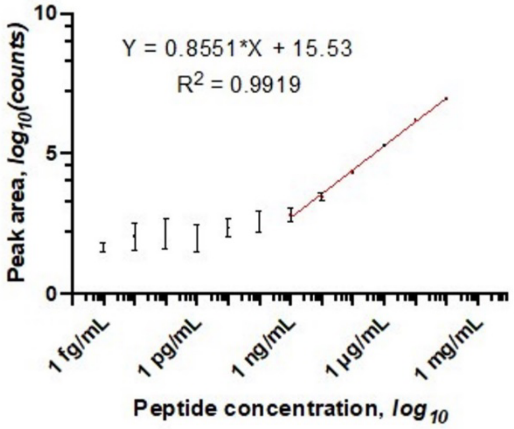

Peptide 10*

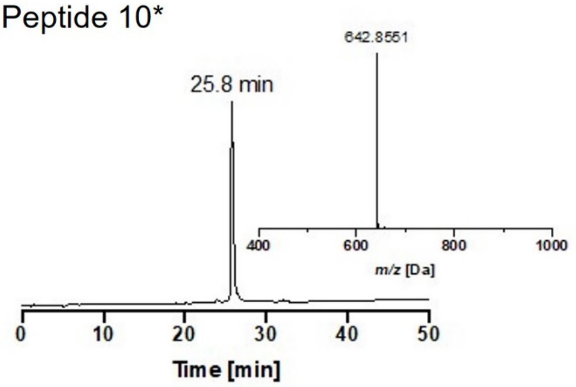

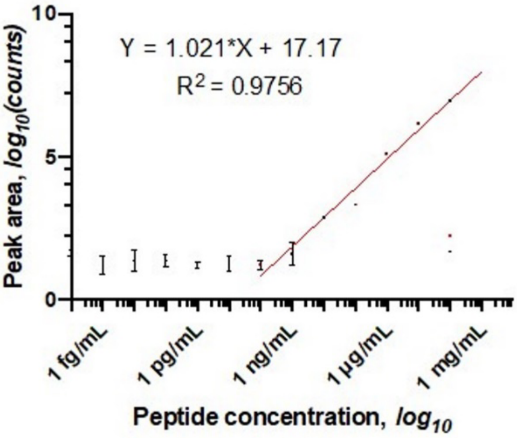

Peptide 10

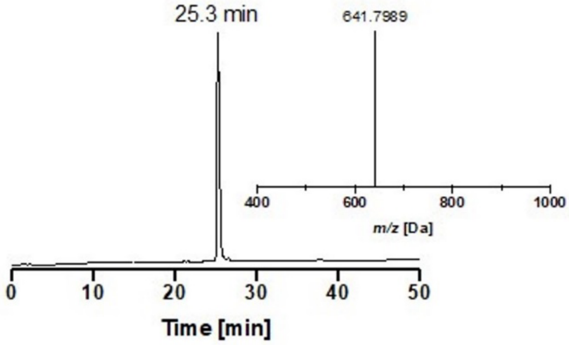

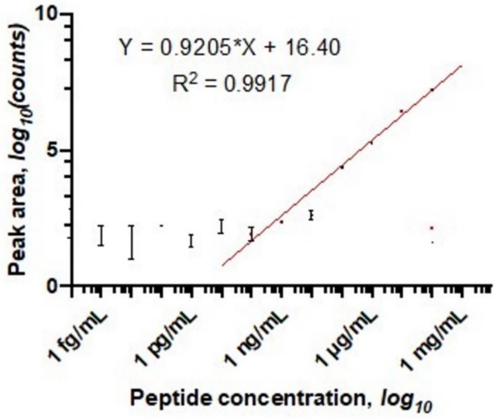

Peptide 11*

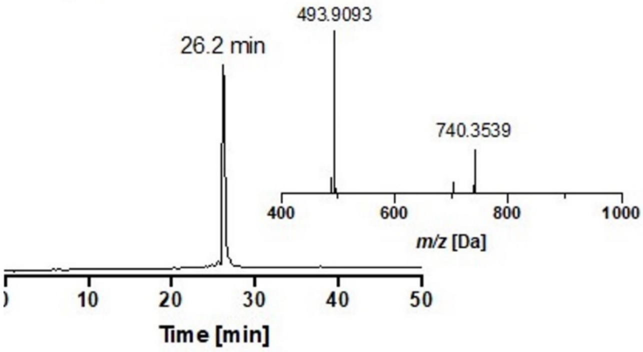

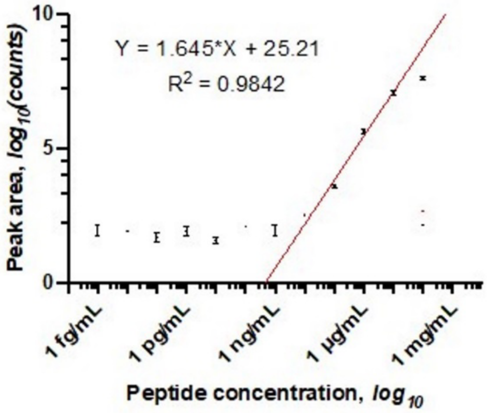

Peptide 11

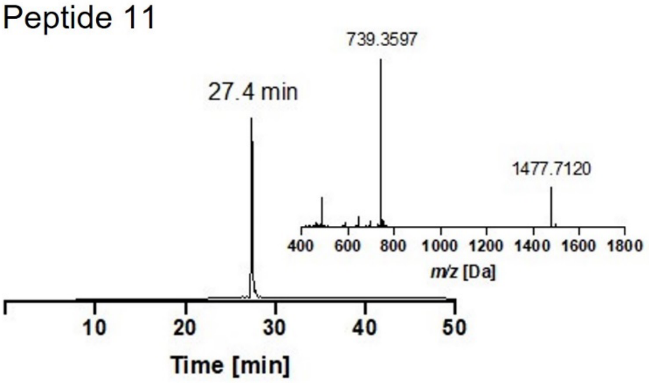

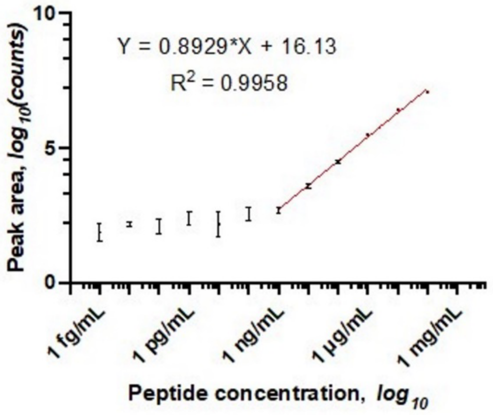

Peptide 12*

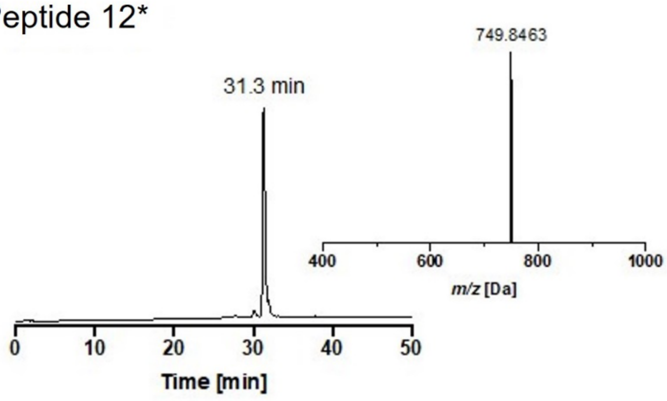

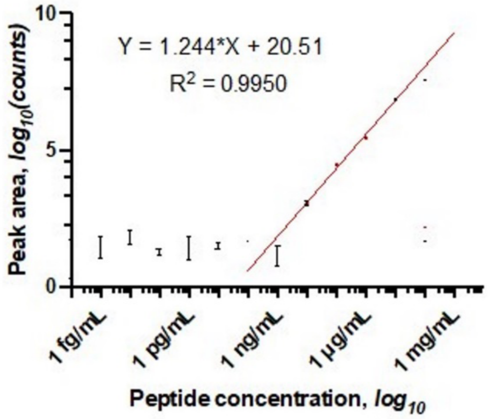

Peptide 12

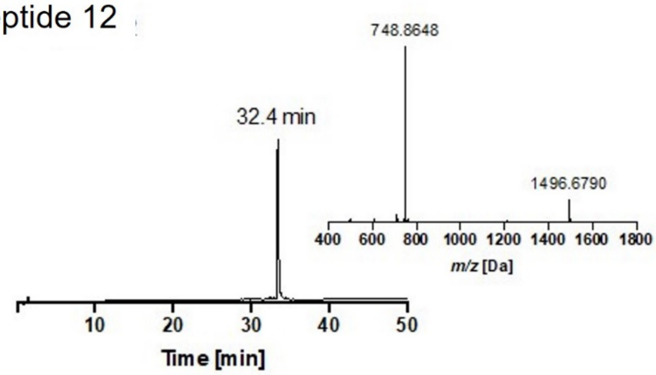

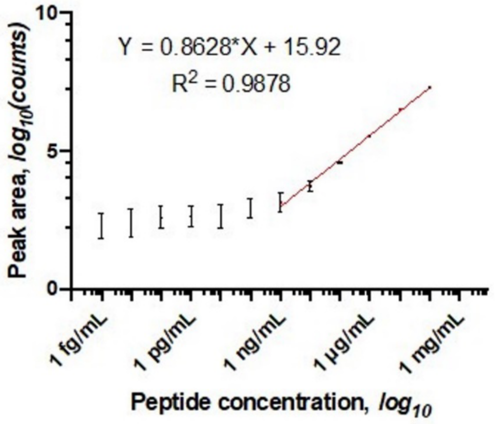

Peptide 13*

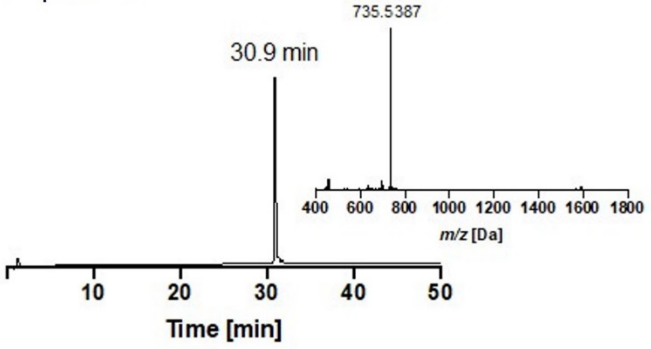

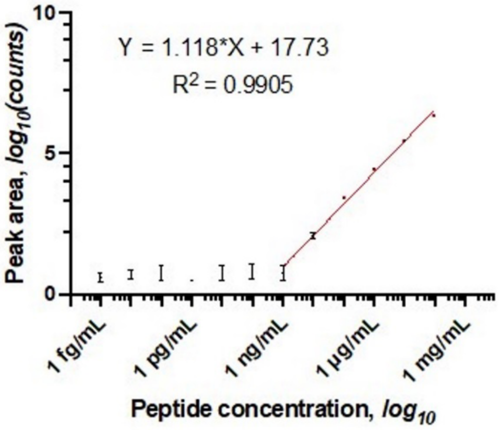

Peptide 13

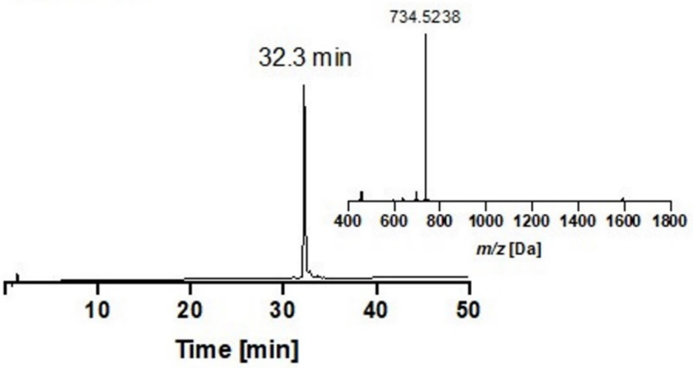

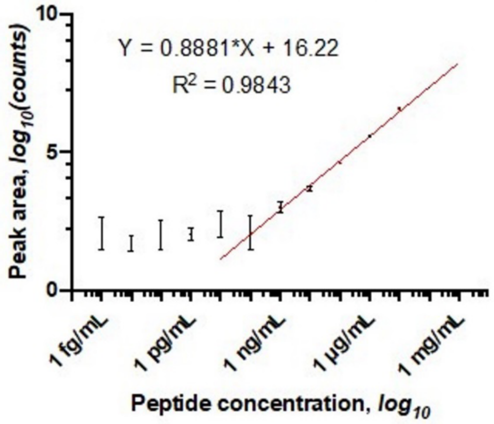

Peptide 14*

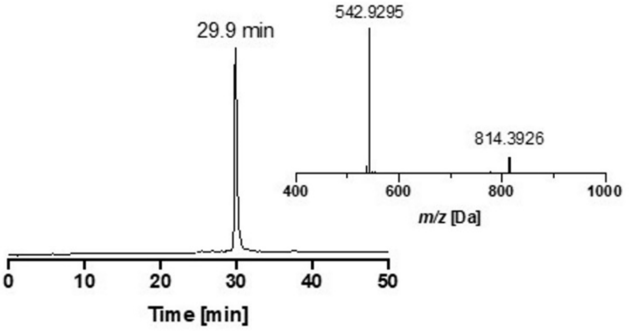

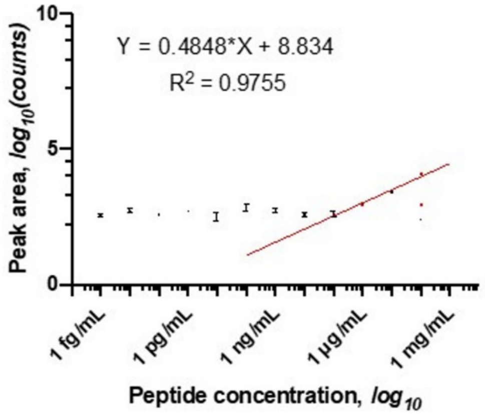

Peptide 14

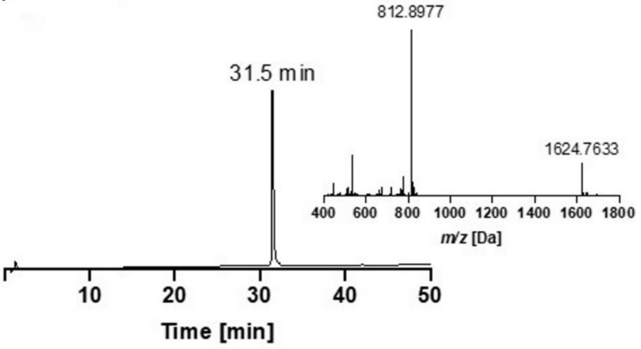

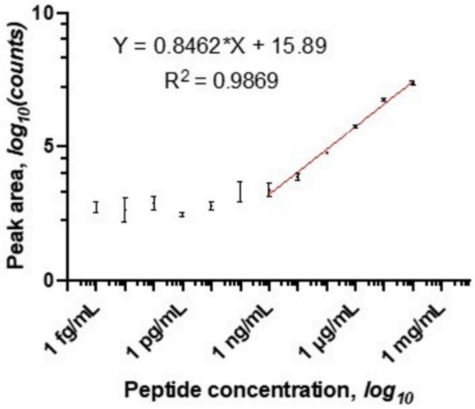

Peptide 15*

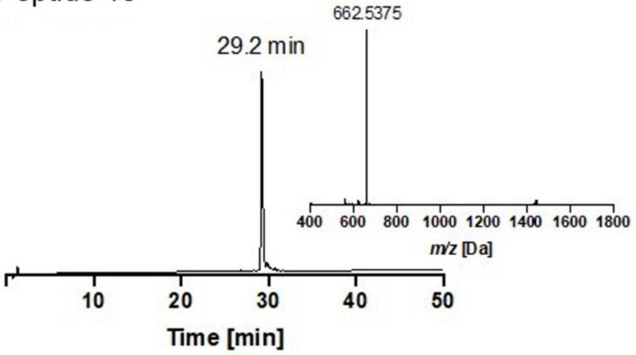

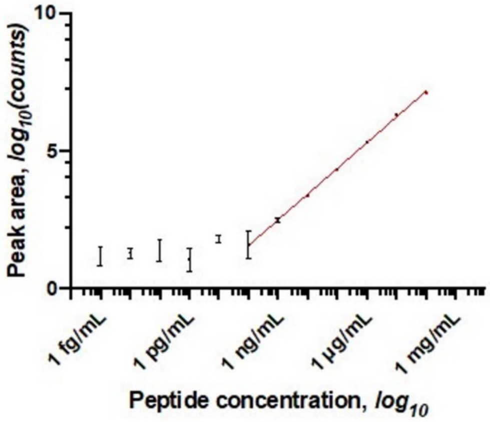

Peptide 15

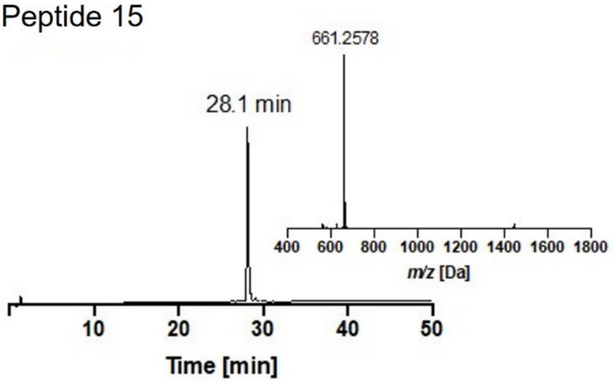

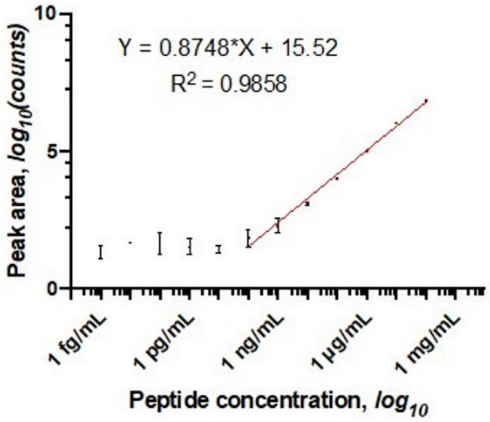

Peptide 16*

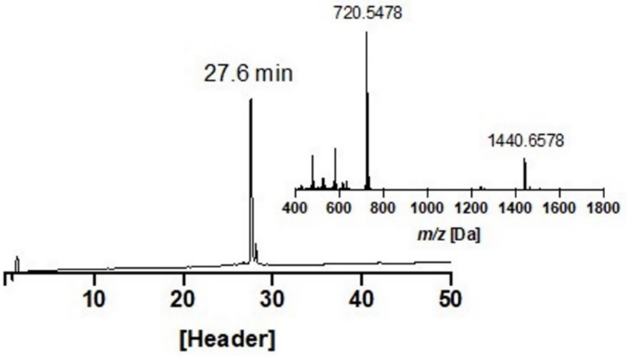

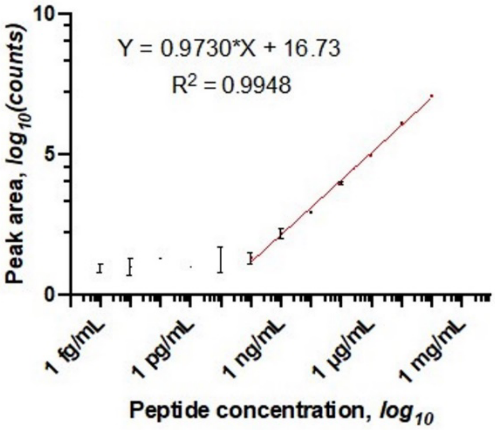

Peptide 16

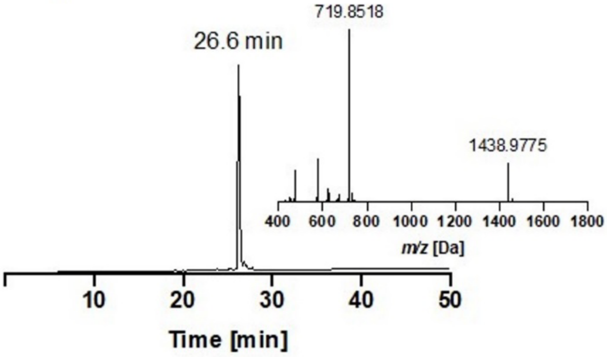

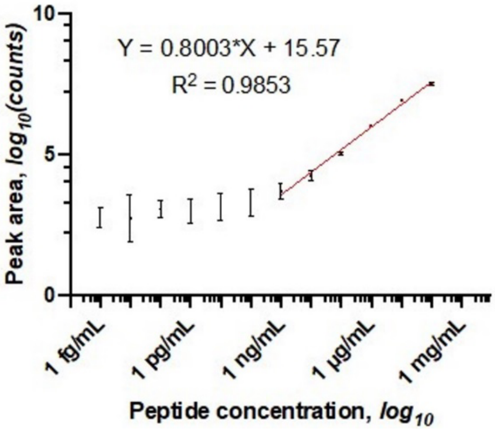

Peptide 17

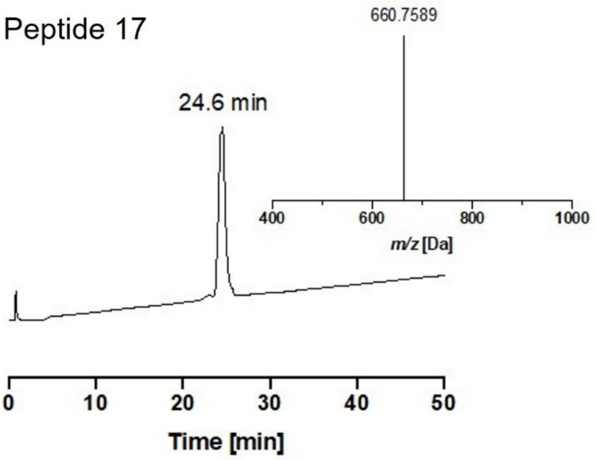

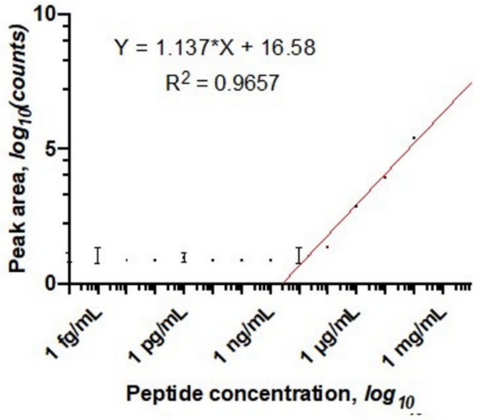

Peptide 18

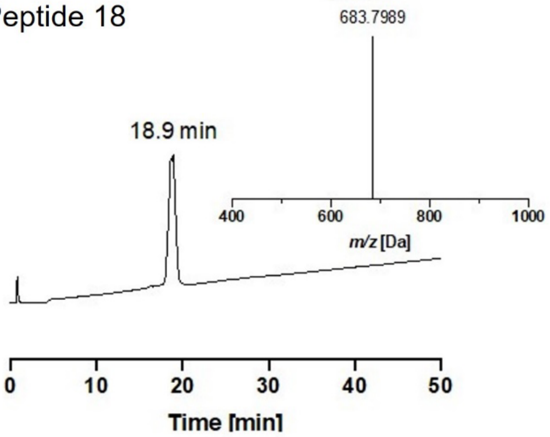

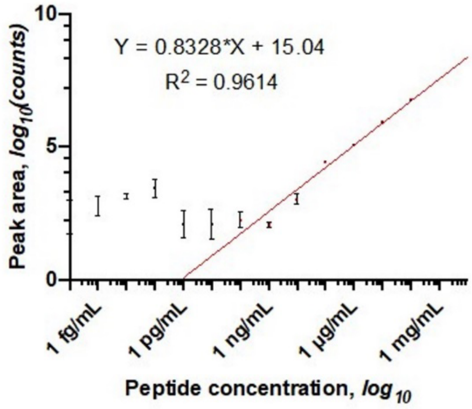

Peptide 19

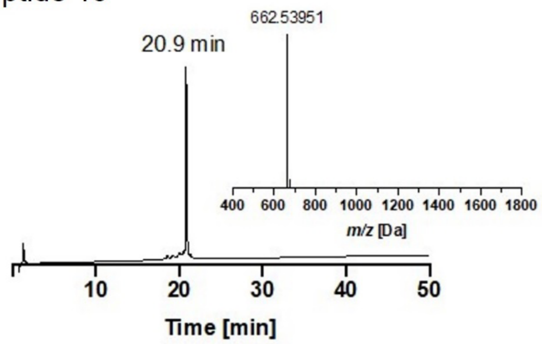

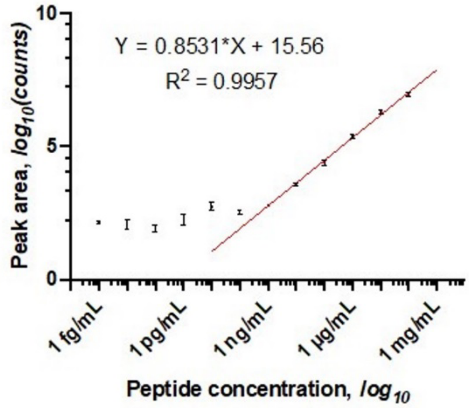

Peptide 20

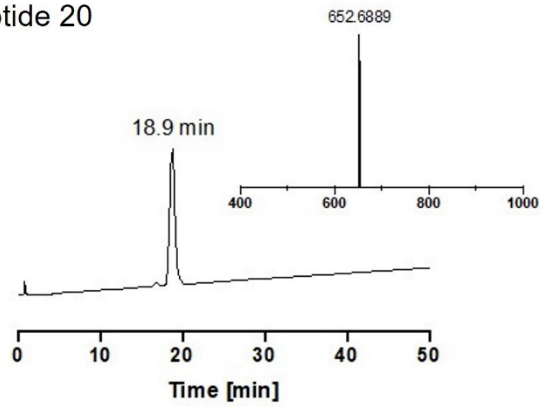

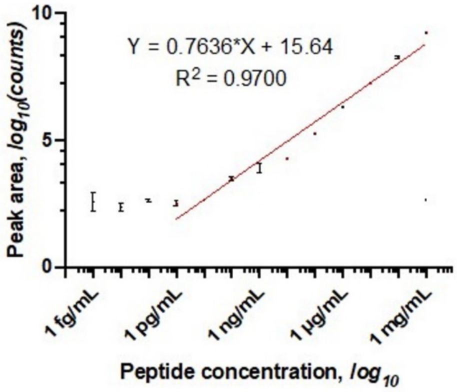

3.4 NMR Spectra

4-bromo-N,N,N-triethylethan-1-aminium (i)

^1H NMR (400 MHz, D_2O) δ 1.29 (t, 9H, J = 7.4 Hz, H6, H7 and H8) 1.87 (dt, 2H, J = 14.0, 6.9 Hz, H9), 1.95 (q, 2H, J = 6.6, 6.6, 6.2, 4.5 Hz, H10), 3.21 (h, 2H, J = 5.9, 4.1, 3.5, 3.1 Hz, H2), 3.31 (q, 6H, J = 7.3 Hz, H3, H4 and H5), 3.57 (t, 2H, J = 6.2 Hz, H11).

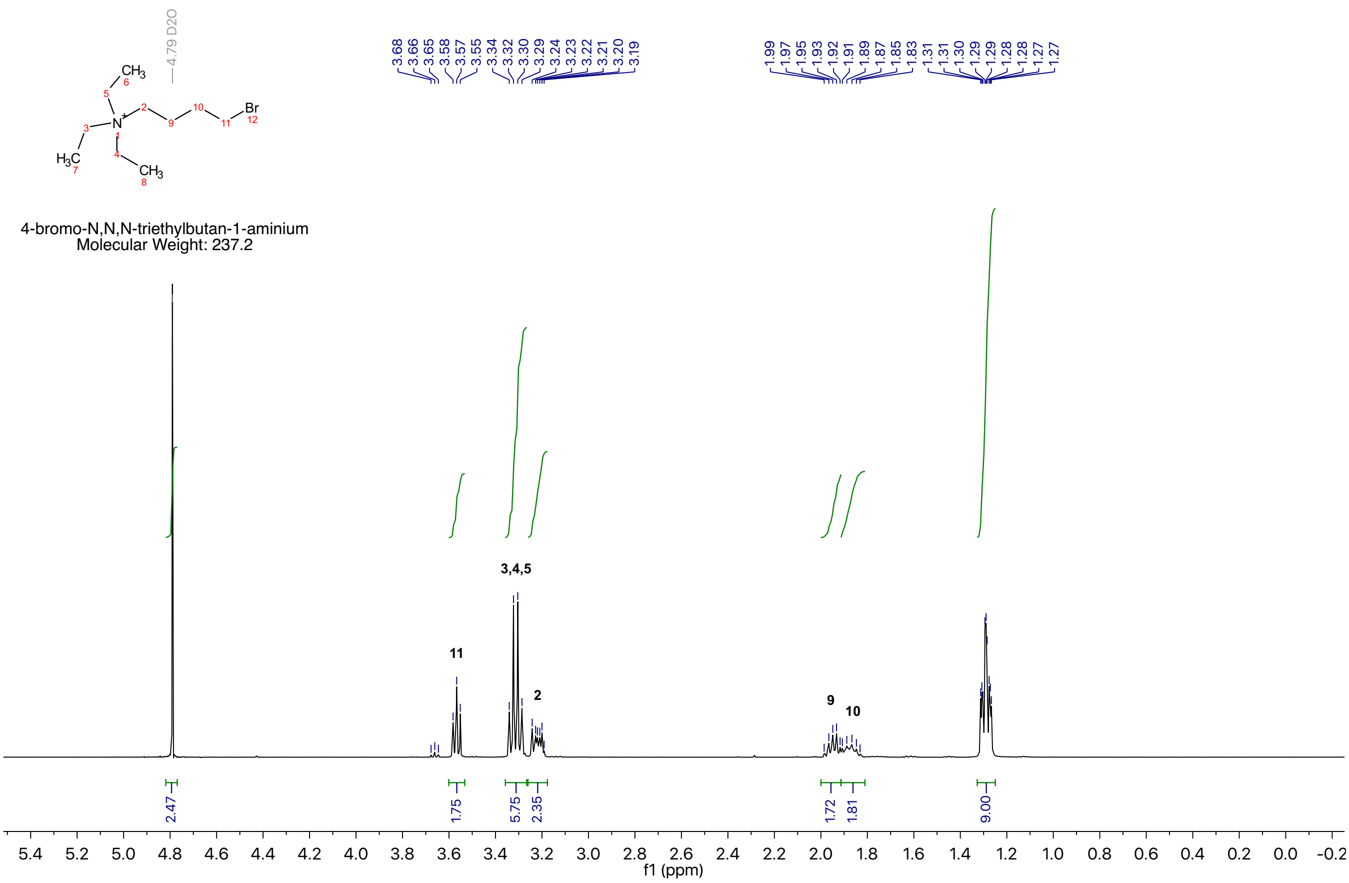

3-(4-bromobutyl)-1-butyl-1*H*-imidazol-3-ium (ii)

^{1}H NMR (400 MHz, D_2O) δ 0.93 (t, 3H, J = 7.4 Hz, H9), 1.32 (h, 2H, J = 7.4 Hz, H8), 1.88 (dq, 4H, J = 14.6, 7.3, 6.8 Hz, H7, H12), 2.06 (p, 2H, J = 7.2 Hz, H11), 3.53 (t, 2H, J = 6.4 Hz, H13), 4.22 (t, 2H, J = 7.1 Hz, H6), 4.26 (t, 2H, J = 7.1 Hz, H10), 7.53 (d, 2H, J = 2.1 Hz, H1, H2).

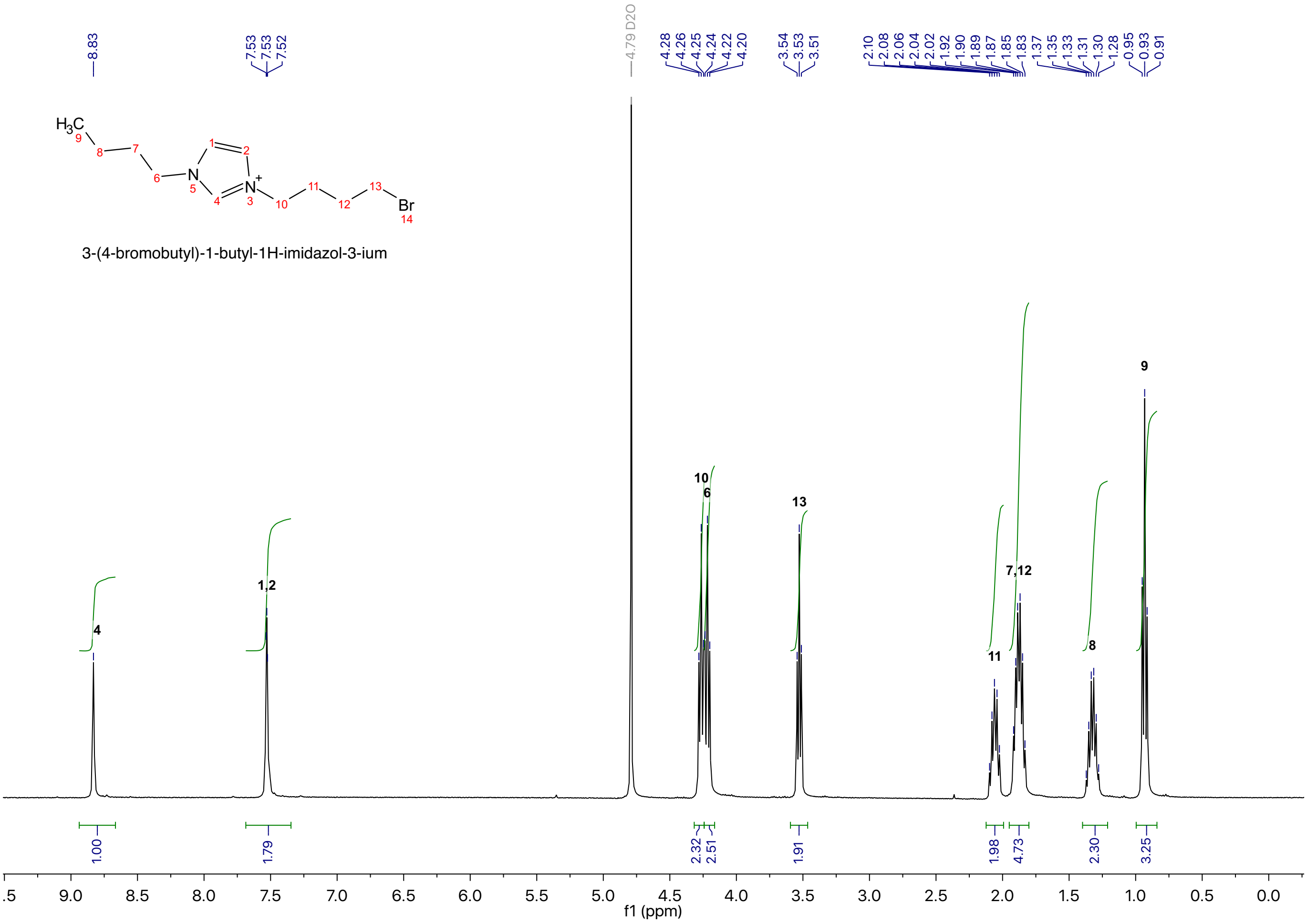

1-(4-bromobutyl)-4-methylpyridin-1-ium (iv)

^{1}H NMR (400 MHz, D_2O) δ 1.93 (p 2H, *J* = 8.5, 6.9, 6.4, 6.4 Hz, H10), 2.17 (p, 2H, *J* = 7.6 Hz, H9), 2.66 (s, 4H, H7), 3.54 (t, 2H, *J* = 6.4 Hz, H11), 4.59 (t, 2H, *J* = 7.4 Hz, H8), 7.89 (d, 2H, *J* = 6.4 Hz, H1, H3), 8.67 (d, 2H, *J* = 6.8 Hz, H4, H6).

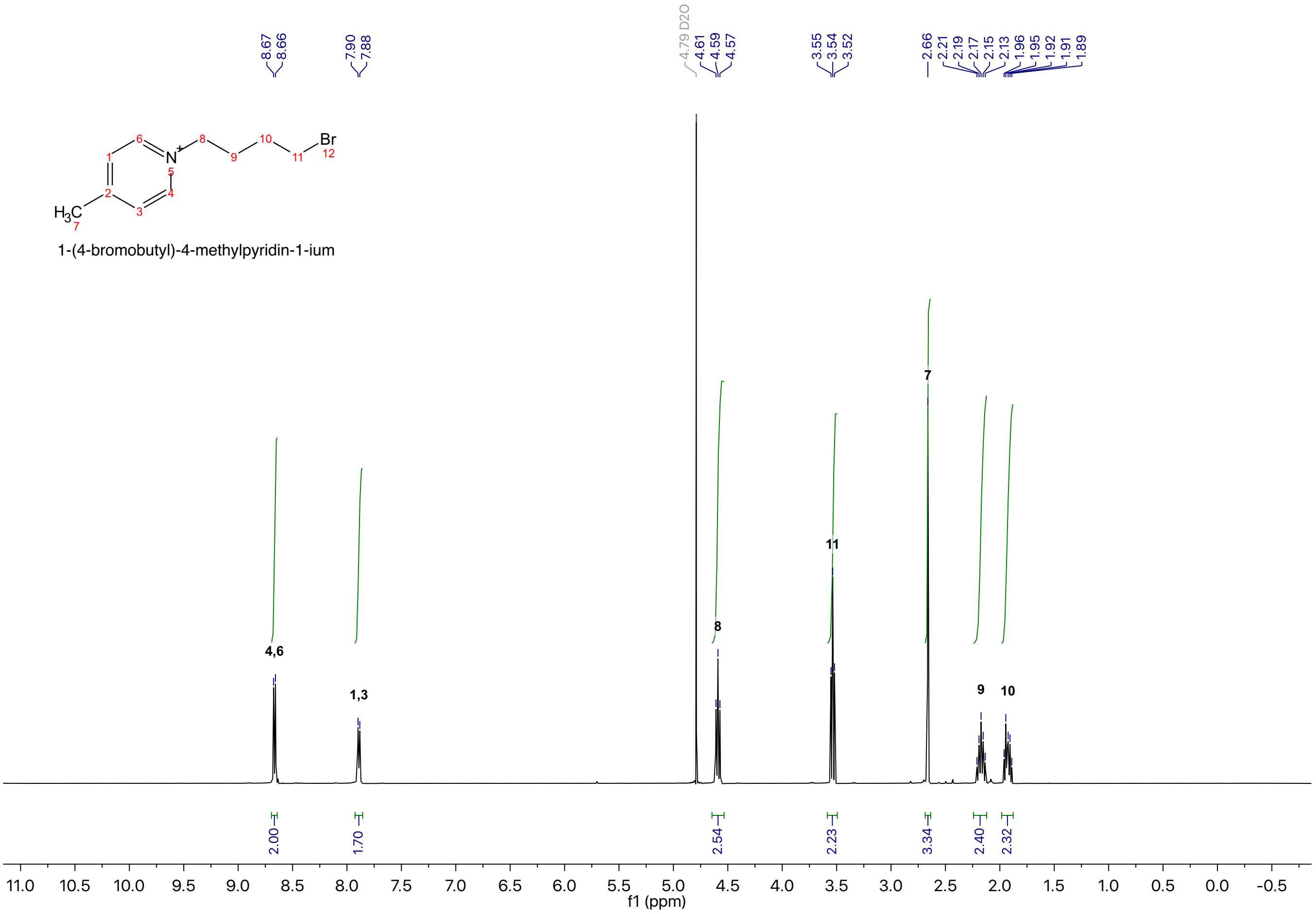

References

ADMINISTRATION, U. F. A. D. 2018. Bioanalytical Method Validation Guidance for Industry.

BUCK, M. A., OLAH, T. A., WEITZMANN, C. J. & COOPERMAN, B. S. 1989. Protein estimation by the product of integrated peak area and flow rate. *Anal. Biochem.,* 182**,** 295-9.

CONIBEAR, A. C., DALY, N. L. & CRAIK, D. J. 2012. Quantification of small cyclic disulfide-rich peptides. *Biopolymers,* 98**,** 518-24.

KREMSMAYR, T. & MUTTENTHALER, M. 2022. Fmoc Solid Phase Peptide Synthesis of Oxytocin and Analogues. *Methods Mol. Biol.,* 2384**,** 175-199.

MATUSZEWSKI, B. K., CONSTANZER, M. L. & CHAVEZ-ENG, C. M. 2003. Strategies for the assessment of matrix effect in quantitative bioanalytical methods based on HPLC-MS/MS. *Anal. Chem.,* 75**,** 3019-30.

MOFFATT, F., SENKANS, P. & RICKETTS, D. 2000. Approaches towards the quantitative analysis of peptides and proteins by reversed-phase high-performance liquid chromatography in the absence of a pure reference sample. *J. Chromatogr. A,* 891**,** 235-42.
